# Microprotein MP104 Promotes Malignant Progression of Colorectal Cancer Through Regulating Protein Translation

**DOI:** 10.1002/advs.76593

**Published:** 2026-07-23

**Authors:** Fang Chen, Miao Wang, Hongmei Yong, Jin Ding, Shiping Xu, Qirui Ge, Yan Wang, Lei Zhang, Qianqian Xiao, Benli Li, Li Lin, Sufang Chu, Qianqing Wang, Jin Bai, Pingfu Hou

**Affiliations:** ^1^ Cancer Institute Cellular Therapeutics School of Medicine Xuzhou Medical University Xuzhou Jiangsu China; ^2^ Department of Laboratory Medicine Affiliated Hospital of Xuzhou Medical University Xuzhou Jiangsu China; ^3^ Medical Technology School of Xuzhou Medical University Xuzhou Jiangsu China; ^4^ Department of Oncology The Affiliated Huai'an Hospital of Xuzhou Medical University and The Second People's Hospital of Huai'an Huai'an Jiangsu China; ^5^ Department of Gastroenterology Affiliated Jinhua Hospital Zhejiang University School of Medicine Jinhua Zhejiang China; ^6^ Department of Pharmacy the Affiliated Hospital of Xuzhou Medical University Xuzhou Jiangsu China; ^7^ Department of Gynecology Oncology Xinxiang Central Hospital The Fourth Clinical College of Xinxiang Medical University Xinxiang Henan China; ^8^ Department of Oncology Huai'an Hospital Affiliated to Yangzhou University and The Fifth People's Hospital of Huai'an Huai'an Jiangsu China; ^9^ Jiangsu Center for the Collaboration and Innovation of Cancer Biotherapy Xuzhou Medical University Xuzhou Jiangsu China

**Keywords:** colorectal cancer, microprotein MP104, protein translation regulation, UBE2O‐AMPKα2‐mTOR‐EIF4B axis, ZEB1‐AS1

## Abstract

Colorectal cancer (CRC) remains a major cause of cancer mortality, necessitating the identification of novel oncogenic drivers. We report the discovery of MP104, a 104‐amino acid microprotein encoded by the long non‐coding RNA ZEB1‐AS1, which is endogenously expressed and upregulated in CRC with strong association to poor prognosis. Functional assays revealed that MP104 promotes CRC cell proliferation, migration, invasion, and metastasis. Mechanistically, MP104 interacts with UBE2O to facilitate AMPKα2 ubiquitination and degradation, thereby activating mTOR signaling. This activation enhances EIF4B phosphorylation and stability, while MP104 further inhibits RNF40‐mediated EIF4B ubiquitination, collectively sustaining translational upregulation. Thus, MP104 drives CRC progression primarily through reprogramming protein translation via the UBE2O–AMPKα2–mTOR–EIF4B axis, establishing it as a key regulator of oncogenic translational control and a promising biomarker and therapeutic target in CRC.

## Graphical abstract

A previously unrecognized microprotein, MP104, encoded by ZEB1‐AS1, emerges as a critical driver of colorectal cancer progression. MP104 links ubiquitin‐mediated protein degradation with translational reprogramming via the UBE2O–AMPKα2–mTOR–EIF4B axis, revealing an unrecognized layer of oncogenic regulation.

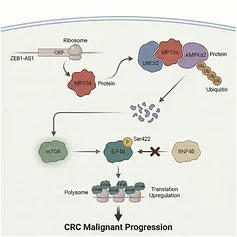

**Abbreviations**
CRC: Colorectal cancer
Co‐IP: co‐immunoprecipitation
CHX: cycloheximide
DSS: disease‐specific survival
GO: Gene ontology
H&E: hematoxylin and eosin
lncRNAs: long non‐coding RNAs
IHC: immunohistochemical
KI: knock‐in
MS: mass spectrometry
ORF: open reading frame
OS: overall survival
PFS: progression‐free survival
DFI: disease‐free interval
smORFs: Small open reading frames
UTRs: untranslated regions
UBE2O: Ubiquitin‐conjugating enzyme E2O

## 1 Introduction

Colorectal cancer (CRC) is one of the most prevalent and lethal malignancies worldwide. An estimated 1.93 million new cases were diagnosed worldwide in 2022, making CRC the third most common cancer, and it accounted for approximately 903 859 deaths, ranking as the second leading cause of cancer mortality [1]. The rising incidence in both developed and developing countries is largely attributed to aging populations, sedentary lifestyles, and westernized diets [2]. Although advances in screening, surgery, chemotherapy, and targeted therapies have improved survival rates, especially in early‐stage disease, the prognosis for patients with advanced or metastatic CRC remains poor, with a 5‐year survival rate <15% [3]. Moreover, resistance to chemotherapy and recurrence after treatment present major clinical challenges, underscoring the urgent need to better understand the molecular mechanisms driving CRC progression and develop novel therapeutic targets.

Small open reading frames (smORFs) are often overlooked in proteome annotation [4]. In recent years, with the advancement of ribosome profiling and mass spectrometry techniques, it has been discovered that many non‐coding RNAs, including lncRNAs, circRNAs, and untranslated regions (UTRs) of mRNAs, can be translated [5, 6]. SmORFs are abundant in many organisms, including the human genome, and some have been found to encode small peptides or microproteins with important biological functions [7]. The open reading frames of some lncRNAs encode microproteins that play significant biological roles [8]. For example, the micropeptide ASAP encoded by LINC00467 promotes CRC progression by directly regulating ATP synthase activity [9]. The micropeptide CIP2A‐BP encoded by LINC00665 can directly bind to the oncogene CIP2A and inhibit the progression of triple‐negative breast cancer [10]. Additionally, a 53‐aa peptide encoded by lncRNA HOXB‐AS3 inhibits CRC growth by blocking hnRNPa1‐mediated PKM splicing and PKM2 formation [11]. The micropeptide pTINCR encoded by lncRNA TINCR inhibits epithelial tumor progression by binding to CDC42, increasing its SUMOylation, and promoting its activation [12]. These studies suggest that non‐coding RNAs may exert their functions by encoding genes, and investigating the coding functions of lncRNAs is of great significance for revealing their roles.

UBE2O (Ubiquitin‐conjugating enzyme E2O) is a unique hybrid ubiquitin enzyme that possesses both E2‐conjugating and E3‐ligase activities, allowing it to catalyze ubiquitination independent of canonical E3 ligases. It plays a critical role in protein quality control by targeting unassembled or misfolded proteins for proteasomal degradation, particularly during erythropoiesis and stress responses [13, 14]. UBE2O has been implicated in the regulation of key signaling pathways, such as by promoting the degradation of AMPKα2, thereby modulating mTOR signaling, which links it to metabolic reprogramming and cancer progression [15]. Recent studies have highlighted UBE2O as an oncogenic factor in various cancers, including liver, lung, and colorectal cancers, where it promotes tumor growth and metastasis by destabilizing tumor suppressor proteins or enhancing oncogenic signaling [15, 16, 17].

LncRNAs have been extensively studied in various cancers, where they are often upregulated and are associated with poor prognosis [18, 19]. However, most of these studies have focused on its function as a non‐coding RNA, with little attention paid to its potential protein‐coding capacity. In this study, we aimed to explore the possibility that ZEB1‐AS1 encodes a functional microprotein, MP104, and investigate its role in CRC progression. Our study not only provides novel insights into the coding potential of lncRNAs but also uncovers a previously unrecognized mechanism by which MP104 promotes CRC malignancy through the UBE2O‐AMPKα2‐mTOR‐EIF4B axis. This study has the potential to reshape our understanding of lncRNA function and offer new therapeutic strategies for CRC.

## 2 Methods

### 2.1 Antibodies and Reagents

Primary antibodies for western blotting include anti‐Flag (20543‐1‐AP, Proteintech; 66008‐4‐Ig, Proteintech), anti‐Myc (AE070, Abclonal), anti‐EIF4B (A13300, Abclonal), anti‐S6 (A11874, Abclonal), anti‐p‐S6‐S235/236 (AP0583, Abclonal), anti‐Puromycin (A21205, Abclonal), anti‐RPL35A (A17938, Abclonal), anti‐RPL8 (A10042, Abclonal), anti‐RPL30 (17403‐1‐AP, Proteintech), anti‐RPS27A (A2027, Abclonal), anti‐EIF1AX/Y (A4270, Abclonal), anti‐RPL10A (16681‐1‐AP, Proteintech), anti‐UBE2O (15812‐1‐AP, Proteintech), anti‐NAP1L1 (A6174, Abclonal), anti‐AMPKα2 (18167‐1‐AP, Proteintech), anti‐p‐EIF4B‐S422 (82550‐1‐RR, Proteintech), anti‐GAPDH (60004‐1‐Ig, Proteintech), anti‐His (10001‐0‐AP, Proteintech), anti‐HA (51064‐2‐AP, Proteintech), anti‐Ubiquitin (#3933, Cell Signaling Technology), anti‐mTOR (#2972, Cell Signaling Technology), anti‐p‐mTOR (#2971, Cell Signaling Technology), anti‐P70‐S6K (#9202, Cell Signaling Technology), anti‐P‐P70‐S6K (#9205, Cell Signaling Technology), anti‐Ki67 (A20018, Abclonal), Anti‐ALB (SC‐271605, Santa Cruz Biotechnology, Inc), anti‐FLAG Magnetic Beads (HY‐K0207, MedChemExpress), anti‐HA magnetic beads (HY‐K0201, MedChemExpress), anti‐c‐Myc Magnetic Beads (HY‐K0206, MedChemExpress), Rapamycin (AY‐22989, MedChemExpress), MG132 (HY‐13259, MedChemExpress), Cycloheximide (HY‐12320, MedChemExpress), Protein A/G Magnetic Beads (HY‐K0202, MedChemExpress), jetPRIME (101000046, Polyplus). The MP104 antibody and His‐MP104 fusion protein were custom‐produced and evaluated by Hangzhou HuaAn Biotechnology Co., Ltd.

### 2.2 Cloning and Genetic Constructs

The full‐length sequence of ZEB1‐AS1 (NR_148977.1) was synthesized by Genewiz and subcloned into the pCDH‐CMV‐MCS‐EF1‐GreenPuro plasmid. MP104 expression plasmids containing the 5'UTR and MP104 ORF were cloned into pCDH‐CMV‐MCS‐EF1‐GreenPuro and pCDH‐CMV‐MCS‐EF1‐blast, with a 3×FLAG tag appended to the 3' end of the ORF. The MP104‐mut and ZEB1‐AS1‐mut plasmids were generated by mutating the ATG start codon to TTG using a QuickMutation kit (D0206, Beyotime). The pCMV‐UBE2O‐3×Myc‐Neo, pCMV‐UBE2O (1‐903aa)‐3×Myc‐Neo, pCMV‐UBE2O (904‐1292aa)‐3×Myc‐Neo, pLV3‐CMV‐EIF4B‐3×HA‐Blast, pLV3‐CMV‐EIF4B‐S422A‐3×HA‐Blast, pCMV‐PRKAA2 (AMPKα2)‐3×HA‐Neo, pCMV‐3×Myc‐RNF40‐Neo, pCMV‐Parkin‐3×Myc‐Neo, pCMV‐SMURF1‐3×Myc‐Neo, pEnCMV‐USP11‐3×Myc, pCMV‐HERC5‐3×Myc‐Neo plasmids were cloned and purchased from MiaoLing Plasmid Platform. The sequences of MP104 are provided in the Supporting Methods section.

### 2.3 Lentivirus Production and Stable Cell Line Construction

Lentivirus production was performed as previously described [20]. Briefly, 293T cells were co‐transfected with the expression or knockout constructs in pCDH‐CMV‐MCS‐EF1‐GreenPuro or pLV‐hCas9‐T2A‐Puro‐U6‐gRNA#1‐U6‐gRNA#2, along with psPAX2 and pMD2.G, using PEI. The culture medium was replaced 12 h after transfection. Forty‐eight hours post‐transfection, the virus‐containing medium was harvested, filtered using a 0.45 µm filter, and then stored at −80°C. Virus‐containing medium was used for stable cell line construction. Twelve hours after infection, the medium was replaced with fresh medium. Forty‐eight hours post‐infection, 2 µg/mL puromycin was added to ensure stable cell line selection. After approximately 7 days of selection, stable cell lines were established and stored.

### 2.4 Generation of MP104 Knockout and Knock‐In Cell Lines

MP104 knockout (KO) and 3×FLAG knock‐in (KI) cell lines were constructed using pLV‐hCas9. The gRNAs targeting the MP104 genomic region (gRNA#1: 5'‐GGGCCAAGGAAAGGGATCGC‐3', gRNA#2: 5'‐GAGTTCCCTTGATGAGGGGA‐3') were inserted into pLV‐hCas9‐T2A‐Puro‐U6‐gRNA#1‐U6‐gRNA#2 for KO and KI. For KO cell generation, lentiviruses were produced and stable cell lines were established as described above. For KI cell generation, a donor plasmid containing an approximately 480 bp homology left‐arm and 480 bp homology right‐arm sequences around the stop codon of MP104, separated by a 3×FLAG tag, was used. The gRNA and donor plasmids were co‐transfected into 293T and HCT116 cells and selected using 2 µg/mL puromycin for 2 days. Flag‐MP104 expression in KI cells was detected using Western blot or immunofluorescence. Donor sequences are provided in the Supporting Methods section.

### 2.5 Immunoprecipitation and Proteomics

For identification of MP104 peptides, HCT116 cell lysates were collected as described above. For immunoprecipitation, 5 µg of MP104 antibody was incubated with Protein A/G magnetic beads at 4°C for 1 h with gentle rotation. The antibody‐bead complex was incubated with the collected cell lysate overnight at 4°C. After incubation, the beads were washed three times with lysis buffer to remove nonspecifically bound proteins. Finally, the bead‐bound complexes were subjected to mass spectrometry (MS) to identify MP104 peptides. For identification of MP104‐associated proteins, HCT116 cells stably expressing control vector and Flag‐MP104 were used, and cells were cultured and lysed as described above. Three biological replicates were performed in the IP assays, anti‐FLAG Magnetic Beads were used for immunoprecipitation in the HCT116‐Con and HCT116‐Flag‐MP104 cells. Finally, the bead‐bound complexes were subjected to MS analysis to identify Flag‐MP104 binding proteins by Shanghai Bioprofile Co., Ltd. Mass spectrometry data were filtered using a peptide‐spectrum match false discovery rate (PSM FDR) ≤ 0.01 and a protein false discovery rate (Protein FDR) ≤ 0.01 as the criteria for peptide, modification site, and protein identification. Differential analysis of mass spectrometry data was subsequently performed based on the intensity values of proteins bound in the Con and Flag‐MP104 groups. Proteins with a *p*‐value < 0.05 and an absolute fold change (|FC|) > 2 were considered significantly differentially enriched binding proteins.

### 2.6 Western Blot, CCK‐8, Transwell, and Colony Formation Assays

Western blotting, CCK‐8, Transwell, and colony formation assays were performed as previously described [21].

### 2.7 Animal Work

Female BALB/c nude mice (6 weeks old) were purchased from Beijing Vital River Laboratory Animal Technology Co., Ltd. (Beijing, China). All animal experiments were approved by the Animal Care and Use Committee of Xuzhou Medical University. HCT116 and DLD1 cells stably expressing MP104 and Vector were used for the subcutaneous tumorigenesis assays. A total of 5 × 10^6^ cells from the vector control group and MP104 overexpression group were injected subcutaneously into the flank of each mouse, respectively. HCT116 cells stably expressing vector control or MP104 knockout were used for subcutaneous tumorigenesis assays to evaluate the effect of MP104 deletion on tumor growth. Mice were randomly assigned into four groups: Control (Con + Vehicle), MP104 Knockout (KO + Vehicle), Control + Rapamycin (Con + Rapamycin), and MP104 Knockout + Rapamycin (KO + Rapamycin). A total of 1 × 10^7^ cells were subcutaneously injected into the flank of each mouse. For the treatment model, Rapamycin was dissolved in a vehicle containing 5% PEG300, 5% Tween‐80, and 4% ethanol and administered at a dose of 4 mg/kg according to the indicated treatment schedule. Tumor volume (V) was measured every 3 days using calipers and calculated according to the following formula: V = (L × W^2^)/2, where L is the length (longest diameter), and W is the width (shortest diameter) of the tumor. For the lung metastasis model, 1 × 10^6^ HCT116 cells stably expressing either MP104 or the control vector were injected into the tail vein of the mice. After 8 weeks, the mice were sacrificed. The lungs were excised, fixed in 4% paraformaldehyde, and stained with hematoxylin and eosin (H&E) staining. Metastatic nodules of each lung were counted.

### 2.8 Statistical Analysis

Statistical analyses were performed using GraphPad Prism version 9. The associations between ZEB1‐AS1 or MP104 and the clinicopathological parameters of patients with CRC were assessed using the chi‐square test. The Kaplan–Meier method with the log‐rank test was employed to evaluate the correlation between ZEB1‐AS1 expression and patient survival in CRC. The unpaired *t*‐test was used to determine the statistical significance of the differences between groups, one‐way ANOVA or two‐way ANOVA analysis was used to compare the differences between more than two groups. Data are presented as the mean ± standard deviation (SD). Statistical significance was set at *p* < 0.05.

## 3 Results

### 3.1 ZEB1‐AS1 Encodes a Functional Microprotein, MP104

To identify functional lncRNAs with protein‐coding potential, we first screened the GEO dataset (GSE40967 [22]) and identified 44 lncRNAs that are upregulated in colorectal cancer (CRC) tissues (Table S1). Further filtering using the GSCA database (https://guolab.wchscu.cn/GSCA/) yielded seven lncRNAs consistently upregulated across multiple CRC datasets. Among these, four lncRNAs were predicted to possess coding potential based on the OpenProt database analysis (Figure 1A). Survival analysis using GSCA indicated that ZEB1‐AS1 showed the strongest negative correlation with patient prognosis in CRC (Figure 1B). Previous studies have reported the high expression of ZEB1‐AS1 in various cancers and its role in promoting tumor progression [19]; however, these investigations have focused exclusively on its function as a non‐coding RNA. Given its potential to encode a peptide, we selected ZEB1‐AS1 for an in‐depth analysis of its coding ability and biological function. According to OpenProt, ZEB1‐AS1 was predicted to encode a 104‐amino acid microprotein, which we named MP104 (Figure 1C). Evidence from ribosome profiling (Ribo‐seq) further supports the translation of this open reading frame (Figure 1D). To experimentally validate MP104 expression, we generated a specific antibody by immunization with recombinant His‐tagged MP104 (Figure S1). Using this antibody in immunoprecipitation followed by mass spectrometry, we identified three unique MP104‐specific peptide fragments, providing direct evidence of endogenous MP104 expression (Figure 1E).

**FIGURE 1:**
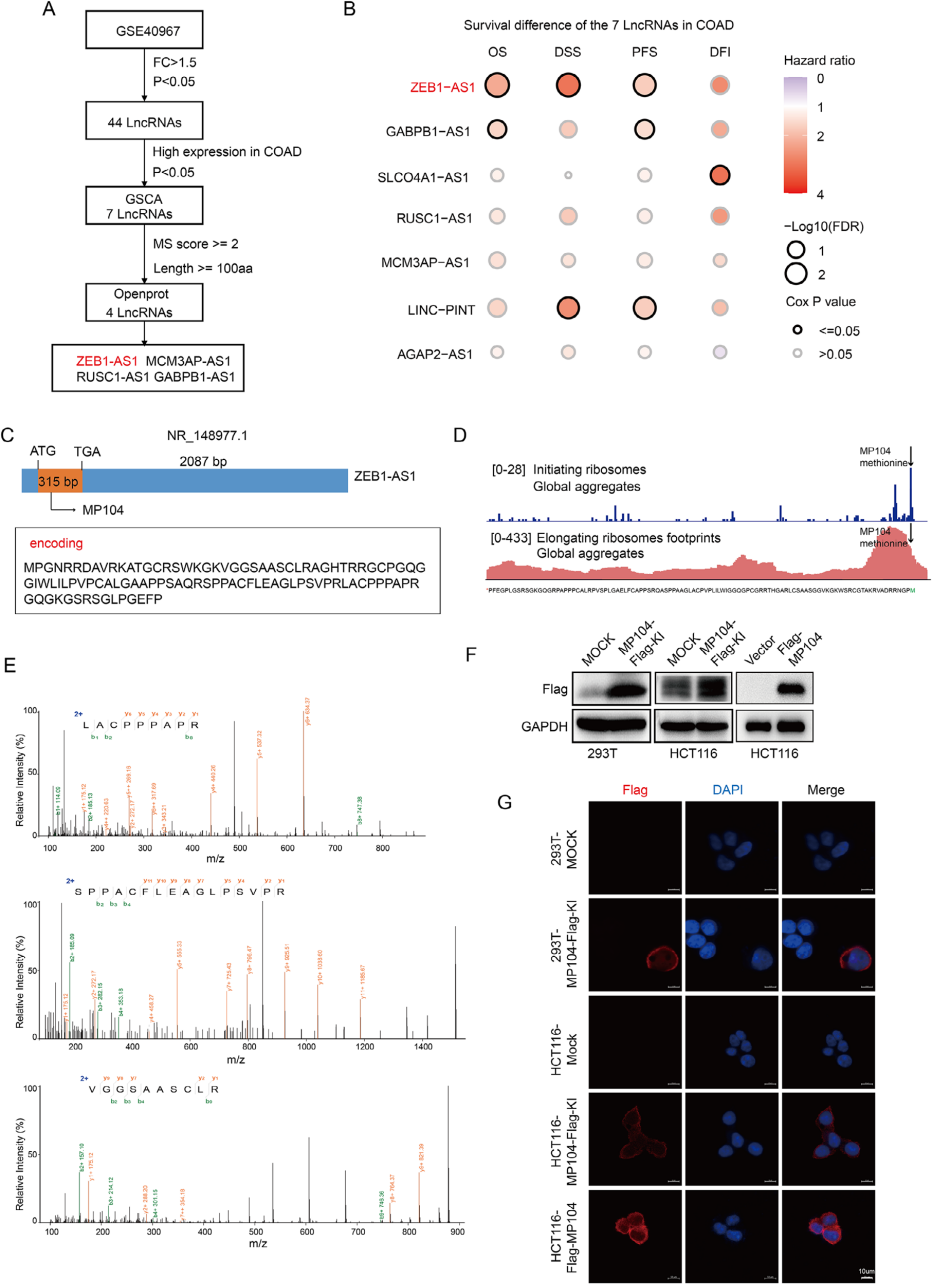
Identification of MP104 encoded by ZEB1‐AS1. (A) Screening process for protein‐coding lncRNAs that are highly expressed in CRC. (B) Analysis of the correlation between the seven lncRNAs—identified through dual screening using the GSE40967 dataset and the GSCA database as highly expressed in CRC—and survival outcomes in COAD patients based on the GSCA database (OS: Overall Survival; DSS: Disease‐Specific Survival; PFS: Progression‐Free Survival; DFI: Disease‐Free Interval). (C) Top: Schematic representation of the ZEB1‐AS1 transcript variant 4 (NR_148977.1), highlighting the open reading frame (ORF) encoding the microprotein MP104. Bottom: Amino acid sequence of MP104. (D) Ribo‐seq data for the ZEB1‐AS1 gene obtained from the GWIPS portal. Initiating ribosome reads are represented by blue bars, while elongating ribosome footprints are shown as a red curve. The start codon (methionine) of MP104 is marked by black arrows. (E) LC‐MS/MS spectra of three tryptic peptides derived from MP104, identified through mass spectrometry analysis following immunoprecipitation of MP104 in HCT116 cells. (F) Detection of the Flag signal by Western blot in MP104‐Flag knock‐in HEK 293T cells, MP104‐Flag knock‐in HCT116 cells, and MP104‐Flag overexpressing HCT116 cells. (G) Confocal imaging of FLAG knock‐in 293T cells, FLAG knock‐in HCT116 cells, MP104‐Flag overexpressing HCT116 cells, and control cells following immunostaining with anti‐FLAG antibody (red) and DAPI (blue). Scale bar: 10 µm.

To confirm that endogenous MP104 is indeed transcribed from the ZEB1‐AS1 genomic locus, we employed CRISPR/Cas9‐mediated knock‐in to insert a FLAG epitope tag at the 3’ end of the MP104 coding sequence in both HEK293T and HCT116 cells. Western blot analysis revealed a specific anti‐FLAG band at the expected molecular weight, confirming successful tagging and expression (Figure 1F). To further validate the endogenous expression and determine the subcellular localization of MP104, we performed immunofluorescence staining in knock‐in (KI) cells. As shown in Figure 1G, endogenously expressed MP104‐Flag was localized predominantly in the cytoplasm, consistent with the localization pattern observed in cells overexpressing exogenous Flag‐tagged MP104. Together, these results conclusively demonstrated that ZEB1‐AS1 encodes an endogenously expressed, cytoplasm‐localized microprotein, MP104.

### 3.2 MP104 is Upregulated in CRC and Predicts Poor Prognosis

Analysis using the GSCA database identified four candidate lncRNAs (ZEB1‐AS1, MCM3AP‐AS1, RUSC1‐AS1, and GABPB1‐AS1), among which ZEB1‐AS1 showed the strongest association with unfavorable survival in CRC (Figure 1B), whereas the other three demonstrated weaker correlations (Figure S2). Further investigation of ZEB1‐AS1 revealed that its expression is markedly elevated across nine cancer types, including colorectal, esophageal, and lung cancers, as shown in the GSCA database (Figure S3A). This suggests that MP104 is involved in the malignant progression of multiple cancers. Analysis of the GSE40967 dataset showed significantly higher ZEB1‐AS1 expression in CRC tissues compared to normal colorectal tissues (Figure 2A). Moreover, its expression was markedly elevated in high‐grade (Stage III and IV) tumors relative to low‐grade (Stage I) ones (Figure 2B). Survival analysis indicated that high ZEB1‐AS1 expression was significantly associated with reduced overall survival (OS) in patients with CRC (Figure 2C). Consistent findings from the GSCA database also demonstrated elevated ZEB1‐AS1 expression in CRC tissues compared to that in normal tissues (Figure 2D), with higher expression levels in advanced pathological stages (Figure 2E). Further survival analysis revealed that high ZEB1‐AS1 levels were significantly correlated with worse overall survival (OS), disease‐specific survival (DSS), progression‐free survival (PFS), and disease‐free interval (DFI) in CRC patients (Figure 2F–I). Using tissue microarrays, we assessed the protein expression of MP104, encoded by ZEB1‐AS1, and found significantly higher levels in CRC tissues than in adjacent normal tissues (Figure 2J,K). Furthermore, MP104 expression was significantly elevated in higher‐grade tumors, mirroring the RNA expression pattern of ZEB1‐AS1 (Figure 2L). To determine whether MP104 could be detected in serum, we analyzed MP104 expression in serum samples from healthy individuals and patients with CRC. MP104 was detectable in the serum of patients with CRC but was not detected in healthy controls (Figure S3B). These results indicate the potential utility of MP104 as a non‐invasive biomarker for CRC detection. Collectively, these findings indicate that both ZEB1‐AS1 and its encoded microprotein, MP104, are highly expressed in CRC and may serve as potential molecular markers for diagnosis and prognosis.

**FIGURE 2:**
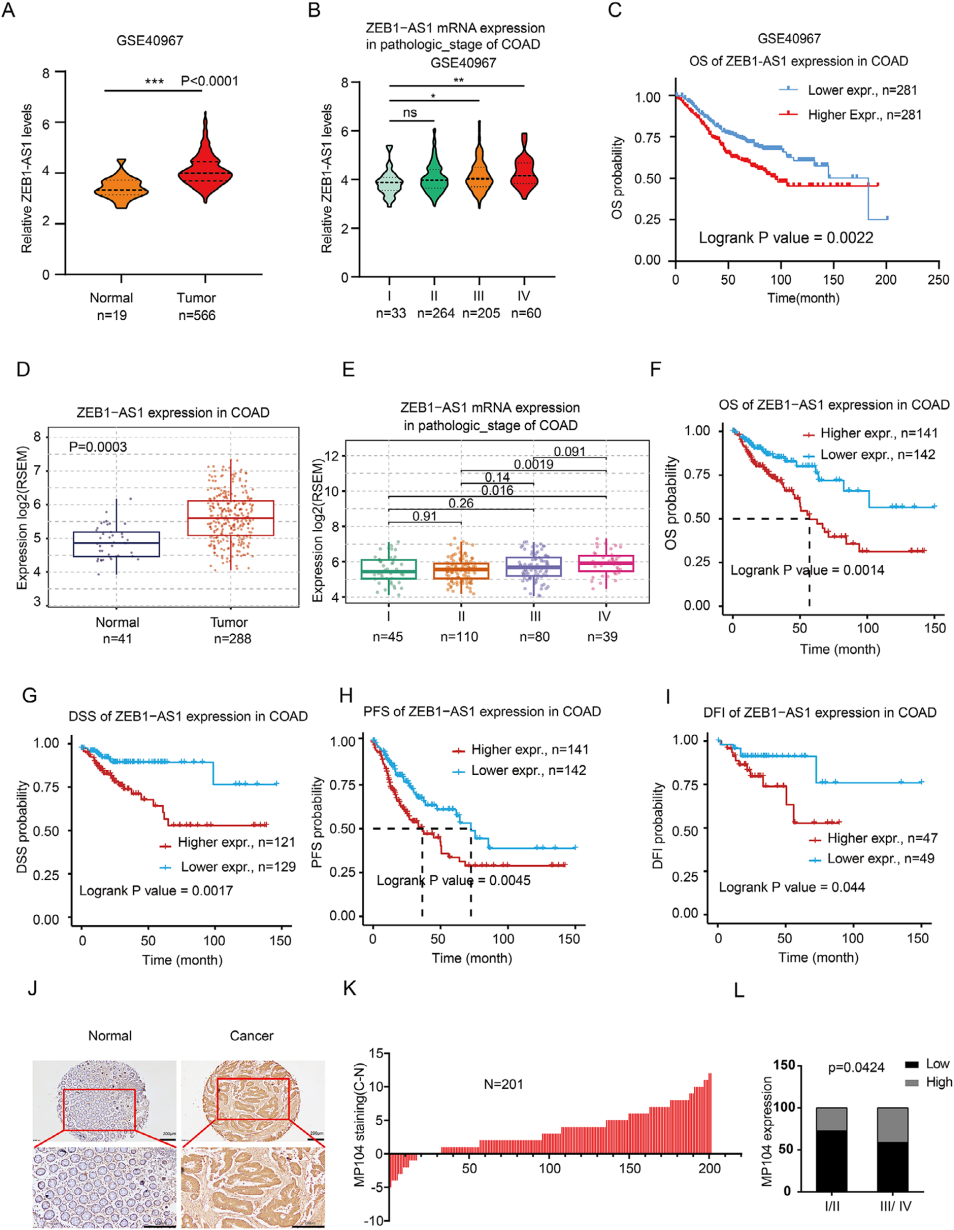
MP104 is upregulated in CRC and its high expression is correlated with poor prognosis. (A) Analysis of ZEB1‐AS1 expression levels in normal intestinal tissues versus colorectal cancer (CRC) tissues based on the GSE40967 dataset. (B) Analysis of ZEB1‐AS1 expression across different CRC stages in the GSE40967 dataset. (C) Correlation between ZEB1‐AS1 expression and overall survival (OS) in CRC patients from the GSE40967 dataset. (D) Differential expression analysis of ZEB1‐AS1 in normal intestinal tissues and CRC tissues using the GSCA database. (E) Analysis of ZEB1‐AS1 expression across CRC stages using the GSCA database. (F–I) Correlation analysis between ZEB1‐AS1 expression and survival outcomes in COAD patients from the GSCA database, including Overall Survival (OS), Disease‐Specific Survival (DSS), Progression‐Free Survival (PFS), and Disease‐Free Interval (DFI). (J) Representative immunohistochemistry (IHC) images showing MP104 expression in colorectal cancer tissues and adjacent normal tissues. (K) Quantification of MP104 staining intensity in CRC tissues compared with paired adjacent noncancerous tissues. N, paired adjacent noncancerous tissues; C, colorectal cancer tissues. (L) Analysis of MP104 expression levels across different stages of CRC based on the IHC analysis.

### 3.3 MP104 Promotes Malignant Features of CRC In Vitro and In Vivo

To explore the function of MP104 in CRC, we measured its expression in CRC and normal epithelial cell lines. The results showed that MP104 was highly expressed in CRC cells compared to normal intestinal epithelial cells (Figure S4A). Given that ZEB1‐AS1 has traditionally been studied as a non‐coding RNA, its protein‐coding potential has largely been overlooked. To determine whether ZEB1‐AS1 functions via its lncRNA activity or through the encoded microprotein MP104, we constructed the following expression plasmids: full‐length ZEB1‐AS1, a start codon mutant (ATG→TTG; ZEB1‐AS1‐Mut), MP104‐Flag, and MP104‐Flag‐Mut (Figure 3A). Stable cell lines expressing these constructs were also generated. Western blot analysis revealed that the overexpression of ZEB1‐AS1 or MP104‐Flag significantly increased MP104 protein levels, whereas the start codon mutants (ZEB1‐AS1‐Mut and MP104‐Flag‐Mut) did not. Deletion the MP104 open reading frame (ORF) significantly reduced MP104 protein expression (Figure 3B). Notably, no significant differences were observed in the relative expression levels of ZEB1‐AS1 mRNA in either the cytoplasmic or nuclear fractions among the MP104, MP104‐Mut, ZEB1‐AS1, and ZEB1‐AS1‐Mut groups. Furthermore, the mutation did not affect the RNA stability of ZEB1‐AS1, indicating that the observed protein differences were not attributable to alterations in RNA stability, or intracellular distribution (Figure S4B,C). Functional assays using CCK‐8 revealed that the overexpression of ZEB1‐AS1 or MP104‐Flag enhanced CRC cell proliferation, whereas the non‐coding mutant ZEB1‐AS1‐Mut had no such effect (Figure 3C,D). Knockdown of MP104 significantly inhibited cell proliferation (Figure 3E,F), while no significant changes were observed in cell cycle progression or apoptosis (Figure S5A,B). Colony formation assays showed consistent results: ZEB1‐AS1 and MP104‐Flag increased colony‐forming ability, whereas ZEB1‐AS1‐Mut did not (Figure S5C,D). MP104 knockdown suppressed colony formation (Figure S5E,F). Transwell assays demonstrated that MP104 and ZEB1‐AS1 promoted cell migration and invasion, whereas non‐coding ZEB1‐AS1‐Mut did not (Figure 3G,H). MP104 knockdown inhibited these effects (Figure 3I,J). These results collectively suggest that ZEB1‐AS1 exerts its oncogenic effects primarily through its encoded microprotein MP104 rather than functioning solely as a lncRNA.

**FIGURE 3:**
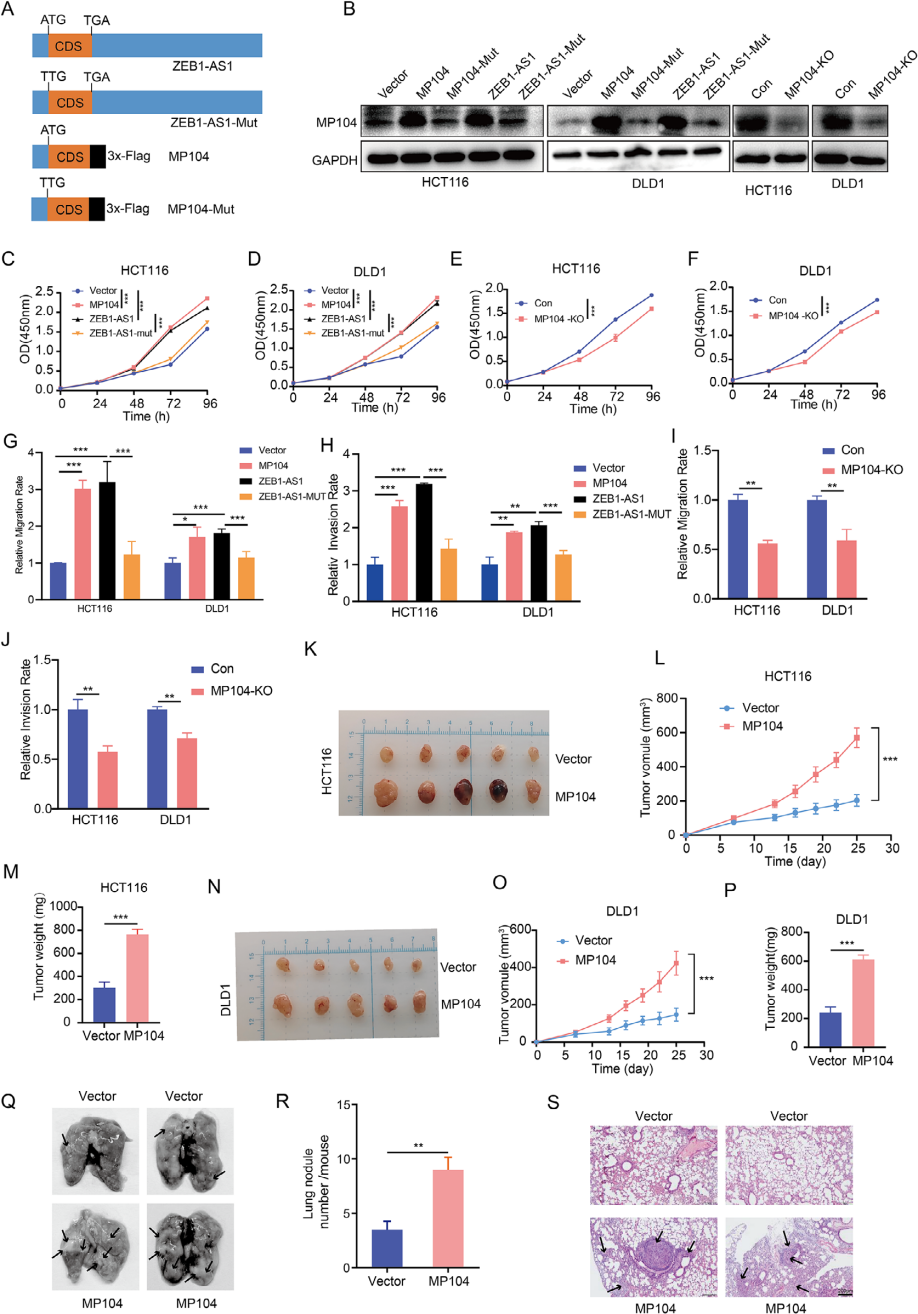
MP104 Promotes Malignant Features of CRC in vitro and in vivo. (A) Diagram of the plasmid constructs. Start codon of ORF was mutated to TTG. (B) Verification of MP104 expression in stably overexpressing and knockout HCT116 and DLD1 cell lines. (C–F) Cell proliferation was assessed using the CCK‐8 assay in the indicated cell lines. Data are presented as mean ± SD from three biologically independent experiments. Statistical analysis was performed using two‐way ANOVA. ***p < 0.001. (G–J) Cell migration and invasion assays were performed to assess the migratory and invasive abilities of the respective cells. Data are presented as mean ± SD from three biologically independent experiments. Statistical significance was determined using unpaired *t*‐tests. **p < 0.01; ***p < 0.001. (K–M) Xenograft mouse model using vector and MP104‐overexpressing HCT116 cells. Representative images of tumors (K), tumor volume (L), and tumor weight (M) are shown. ***p < 0.001. (N–P) Xenograft mouse model using vector and MP104‐overexpressing DLD1 cells. Representative images of tumors (N), tumor volume (O), and tumor weight (P) are shown. ***p < 0.001. (Q–S) Lung metastasis model using vector and MP104‐overexpressing HCT116 cells. Representative images of lungs (Q), lung nodule numbers (R), and HE staining (S) are shown. Scar bar: 200 µm, **p < 0.01.

We further investigated MP104's role in tumorigenesis in vivo. Subcutaneous xenograft models using HCT116 and DLD1 cells showed that overexpression of MP104 significantly accelerated tumor growth (Figure 3K–P). In addition, tail vein injection models revealed that MP104 overexpression significantly promoted lung metastasis in CRC (Figure 3Q–S). These findings demonstrate that MP104 functions as an oncogene in CRC by promoting both tumor growth and metastatic potential, and is closely associated with malignant progression.

### 3.4 MP104 is Involved in the Regulation of Protein Translation

Because many microproteins function by interacting with other proteins to regulate cellular processes, we performed co‐immunoprecipitation (Co‐IP) using Flag‐tagged MP104, followed by quantitative proteomic analysis to identify MP104‐interacting proteins. Mass spectrometry results showed that MP104 binds to UBE2O, as well as its complex components NAP1L4 and NAP1L1 [23], and binds to multiple ribosomal proteins (RPL8, RPL30, RPL35A, RPL10A and RPS27A) and translation initiation factors (EIF1A/Y and EIF4B) (Figure 4A,B). Co‐IP experiments confirmed these interactions (Figure 4C), supporting the mass spectrometry results. Gene Ontology (GO) analysis using the STRING database revealed that MP104‐interacting proteins were enriched in biological processes related to protein translation (Figure 4D), indicating a role of MP104 in translational regulation. To further assess this, we used puromycin incorporation assays to evaluate global protein synthesis. The results showed that MP104 overexpression enhanced protein translation in both 293T and HCT116 cells (Figure 4E,F). Sucrose gradient centrifugation (ribosome profiling) showed that MP104 increased the association of mRNAs with polysomes, suggesting enhanced translational efficiency (Figure 4G).

**FIGURE 4:**
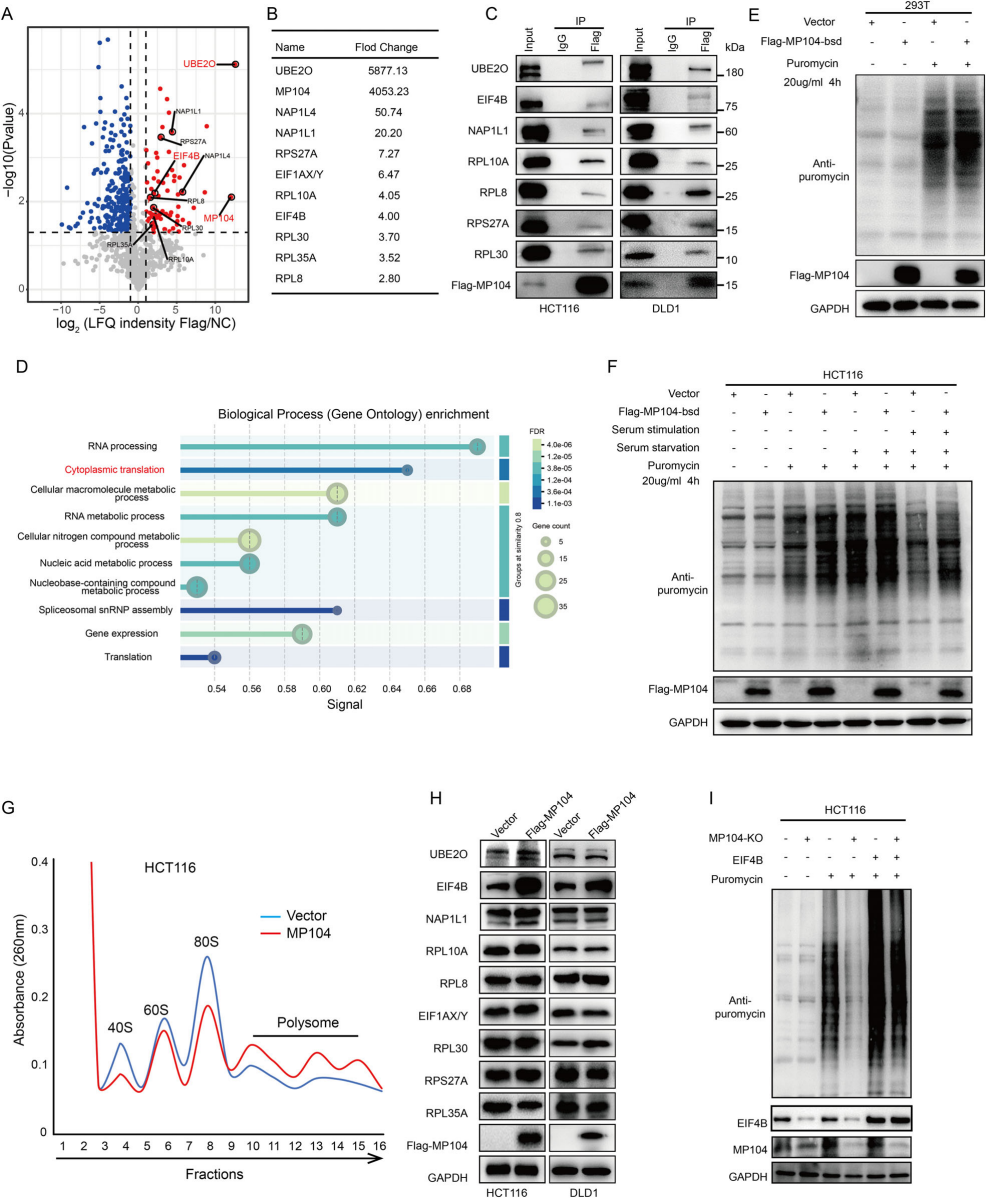
MP104 regulates protein translation. (A) Quantitative proteomics (N = 3) of anti‐Flag magnetic bead pulldown from HCT116 cell lysates overexpressing MP104‐Flag identified MP104‐interacting proteins that were enriched compared to those pulled down with Protein A/G magnetic beads. (B) List of translation‐related proteins identified in the MP104 interactome by mass spectrometry (MS). (C) Co‐immunoprecipitation (IP) analysis of the interactions between Flag‐MP104 and UBE2O, EIF4B, NAP1L1, as well as ribosome‐associated proteins in colorectal cancer cell lines. (D) Gene Ontology (GO) enrichment analysis of the MP104 interactome. (E) Representative western blot images of SUnSET assays quantifying protein synthesis in HEK293T cells expressing either empty vector or MP104. (F) Representative western blot images of SUnSET assays under indicated conditions in HCT116 cells expressing vector or MP104. (G) Polysome profiling of HCT116 cells expressing vector or MP104, assessed by sucrose density‐gradient ultracentrifugation. (H) Western blot analysis of the effects of MP104 overexpression on the expression levels of its binding partners in HCT116 and DLD1 cells. (I) Representative western blot images of SUnSET assays quantifying the effect of EIF4B on protein synthesis in MP104 knockout (KO) HCT116 cells.

Previous studies have shown that UBE2O regulates translation by modulating ubiquitination of ribosomal proteins. To test whether MP104 influenced translation via UBE2O or related proteins, we examined their expression. Interestingly, MP104 specifically upregulated the translation initiation factor EIF4B, without affecting the UBE2O or ribosomal protein levels (Figure 4H). EIF4B is a key component of the eIF complex that facilitates translation initiation [24, 25]. Notably, overexpression of EIF4B in MP104 knockout CRC cells rescued the translation defects, confirming that MP104 enhances protein synthesis by regulating EIF4B expression (Figure 4I).

### 3.5 MP104 Interacts With UBE2O and Enhances UBE2O‐Mediated AMPKα2 Degradation

UBE2O is an E2/E3 ubiquitin‐conjugating enzyme that regulates protein degradation. We first investigated whether EIF4B might be a direct substrate of UBE2O, thereby allowing MP104 to influence EIF4B levels via modulation of UBE2O activity. However, our experiments showed that UBE2O overexpression increased EIF4B protein levels (Figure S6), indicating that EIF4B is unlikely to be a direct ubiquitination substrate of UBE2O. Therefore, we hypothesized that MP104 may indirectly regulate EIF4B by modulating UBE2O activity toward other substrates, which in turn affects the pathways controlling EIF4B stability. Previous studies have reported that UBE2O can bind to AMPKα2 and mediate its ubiquitination and degradation, thereby activating the mTOR pathway [15]. Moreover, activation of mTOR signaling has been shown to enhance EIF4B phosphorylation, thereby stabilizing the EIF4B protein [26, 27]. Based on these findings, we speculated that MP104 might affect UBE2O‐mediated degradation of AMPKα2, leading to mTOR activation, which in turn could modulate EIF4B and ultimately impact global translation.

To test this hypothesis, we first examined whether MP104 physically interacted with UBE2O. Co‐immunoprecipitation assays in cells co‐expressing Flag‐MP104 and Myc‐UBE2O demonstrated reciprocal interaction between MP104 and UBE2O (Figure 5A,B). In addition, pull‐down assays using bacterially expressed His‐MP104 protein confirmed its binding to endogenous UBE2O in cell lysates (Figure 5C). These results verified that MP104 is directly associated with UBE2O. Next, we assessed the effect of MP104 on AMPKα2 protein levels in colorectal cancer cells. Overexpression of MP104 reduced AMPKα2 protein abundance (Figure 5D). Through co‐immunoprecipitation, we observed that MP104 overexpression enhanced the interaction between UBE2O and AMPKα2 and increased AMPKα2 ubiquitination (Figure 5E,F). Moreover, the addition of purified His‐MP104 protein in vitro strengthened the binding between UBE2O and AMPKα2 in the cell lysates (Figure 5G). To confirm that this leads to proteasomal degradation of AMPKα2, we treated the cells with the proteasome inhibitor MG132. MP104's effect on AMPKα2 was abrogated by MG132, indicating that MP104 promotes the proteasomal degradation of AMPKα2 (Figure 5H). Cycloheximide (CHX) chase experiments further showed that MP104 overexpression accelerated the turnover of AMPKα2 via enhanced ubiquitin‐mediated degradation (Figure 5I). Collectively, these data indicate that MP104 facilitates UBE2O's interaction with AMPKα2, thereby promoting AMPKα2 ubiquitination and proteasomal degradation, suggesting that MP104 regulates UBE2O function primarily through direct protein–protein interaction at the post‐translational level rather than through transcriptional or expression‐level regulation.

**FIGURE 5:**
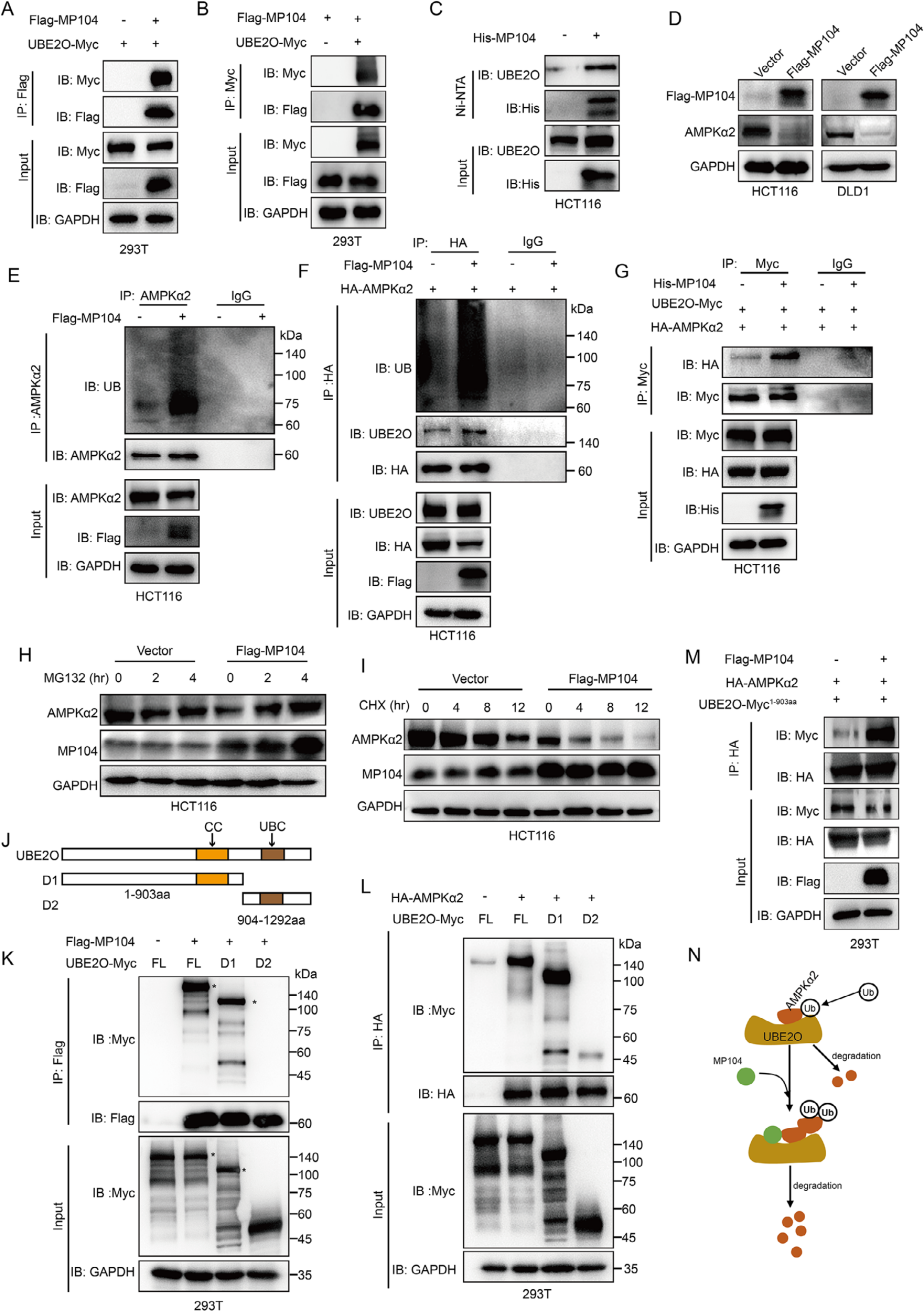
MP104 enhances UBE2O‐Mediated AMPKα2 degradation. (A,B) Flag‐tagged MP104 and Myc‐tagged UBE2O were co‐expressed in HEK293T cells, and co‐immunoprecipitation (Co‐IP) assays were performed to assess the interaction between MP104 and UBE2O. (C) Recombinant His‐tagged MP104 protein was incubated with HCT116 cell lysates, followed by Ni‐NTA pulldown to detect the interaction between MP104 and UBE2O. (D) Western blot analysis of AMPKα2 protein levels in HCT116 and DLD1 cells expressing either empty vector or Flag‐MP104. (E) Lysates from HCT116 cells expressing Flag‐MP104 or vector control were subjected to immunoprecipitation with anti‐AMPKα2 or IgG control, followed by immunoblotting for ubiquitin to assess AMPKα2 ubiquitination. (F) Lysates from HCT116 cells co‐expressing Flag‐MP104 and HA‐AMPKα2 were immunoprecipitated using anti‐HA or IgG control, followed by immunoblotting for ubiquitin or with the indicated antibodies to examine the interaction between UBE2O and AMPKα2. (G) Recombinant His‐MP104 protein was incubated with lysates from HCT116 cells co‐expressing UBE2O‐Myc and HA‐AMPKα2. Immunoprecipitation was performed with anti‐Myc or IgG, and interactions between UBE2O and AMPKα2 were analyzed with the indicated antibodies. (H) Western blot showing the effects of the proteasome inhibitor MG132(10 µM) on AMPKα2 protein accumulation over time in HCT116 cells expressing vector or Flag‐MP104. (I) Western blot showing AMPKα2 protein degradation in HCT116 cells expressing vector or Flag‐MP104 at the indicated time points following cycloheximide (CHX, 100 µg/mL) treatment. (J) Schematic representation of conserved domains in UBE2O and its deletion mutants (D1 and D2). CC: coiled‐coil domain; UBC: ubiquitin‐conjugating domain. (K) HEK293T cells were transfected with Flag‐MP104 and either full‐length (FL) or deletion mutants (D1, D2) of UBE2O‐Myc as indicated. Protein interactions were analyzed by immunoprecipitation (IP) and immunoblotting (IB). (L) HEK293T cells were transfected with HA‐AMPKα2 and either full‐length (FL) or deletion mutants (D1, D2) of UBE2O‐Myc as indicated. Protein interactions were analyzed by IP and IB. (M) HEK293T cells were transfected with Flag‐MP104, HA‐AMPKα2, and UBE2O‐Myc(1–903aa) (D1) constructs as indicated, and protein interactions were analyzed by IP and IB. (N) Schematic model illustrating the regulatory mechanism of the MP104‐UBE2O‐AMPKα2 axis.

To elucidate how MP104 enhances the UBE2O‐AMPKα2 interaction, we performed truncation mapping of UBE2O to identify the domains required for binding to MP104 and AMPKα2. Our results revealed that the coiled‐coil (CC) domain of UBE2O mediates its association with both MP104 and AMPKα2 (Figure 5J–L). Furthermore, MP104 expression specifically enhanced the interaction between the UBE2O CC domain and AMPKα2 (Figure 5M). These findings suggested that MP104 binds to the CC domain of UBE2O, promoting or stabilizing its engagement with AMPKα2. In summary, our data demonstrated that MP104 directly interacts with UBE2O and enhances UBE2O's binding to its substrate AMPKα2 via the UBE2O CC domain, leading to accelerated ubiquitination and proteasomal degradation of AMPKα2 (Figure 5N). This mechanism likely contributes to the downstream activation of mTOR signaling and stabilization of EIF4B, thereby promoting translational activity and oncogenic processes in colorectal cancer.

### 3.6 MP104 Regulates Translation via the UBE2O‐AMPKα2‐mTOR‐EIF4B Axis

UBE2O has been reported to ubiquitinate AMPKα2, leading to its degradation and the subsequent activation of the mTOR pathway [15]. Therefore, we investigated whether MP104 modulated EIF4B expression by influencing the UBE2O‐AMPKα2‐mTOR signaling cascade. In colorectal cancer cell lines, overexpression of MP104 led to a decrease in AMPKα2 protein levels and concomitant activation of mTOR signaling, as evidenced by the increased phosphorylation of S6 and S6K. Moreover, MP104 overexpression enhanced the phosphorylation of EIF4B at Ser422 and increased total EIF4B protein abundance (Figure 6A,B). To assess dependency on UBE2O, we silenced UBE2O in MP104‐overexpressing cells. UBE2O knockdown reversed MP104's effects: AMPKα2 levels were restored, mTOR activation was suppressed (marked by decreased phosphorylation of S6 and S6K), and phosphorylation and total levels of EIF4B were reduced (Figure 6A,B). To further investigate whether AMPKα2 mediates MP104‐induced mTOR activation, we performed rescue experiments by restoring AMPKα2 expression in HCT116‐MP104 cells. Western blot analysis showed that re‐expression of AMPKα2 markedly attenuated MP104‐induced mTOR pathway activation, as evidenced by reduced phosphorylation levels of mTOR, S6K, S6, and EIF4B (Figure S7). Conversely, genetic ablation of MP104 in CRC cells resulted in elevated AMPKα2 expression, inhibition of mTOR signaling (decreased phospho‐S6 and phospho‐S6K), and reduced EIF4B phosphorylation and protein levels (Figure 6C,D). These findings indicated that endogenous MP104 supports mTOR pathway activation by promoting AMPKα2 degradation. To confirm that MP104's effect on EIF4B requires mTOR activity, we treated the cells with rapamycin to inhibit mTOR. Under rapamycin treatment, MP104 overexpression did not enhance EIF4B phosphorylation or increase its protein levels (Figure 6E). This result indicates that MP104 regulates EIF4B via mTOR‐dependent mechanisms rather than through the direct modulation of EIF4B stability.

**FIGURE 6:**
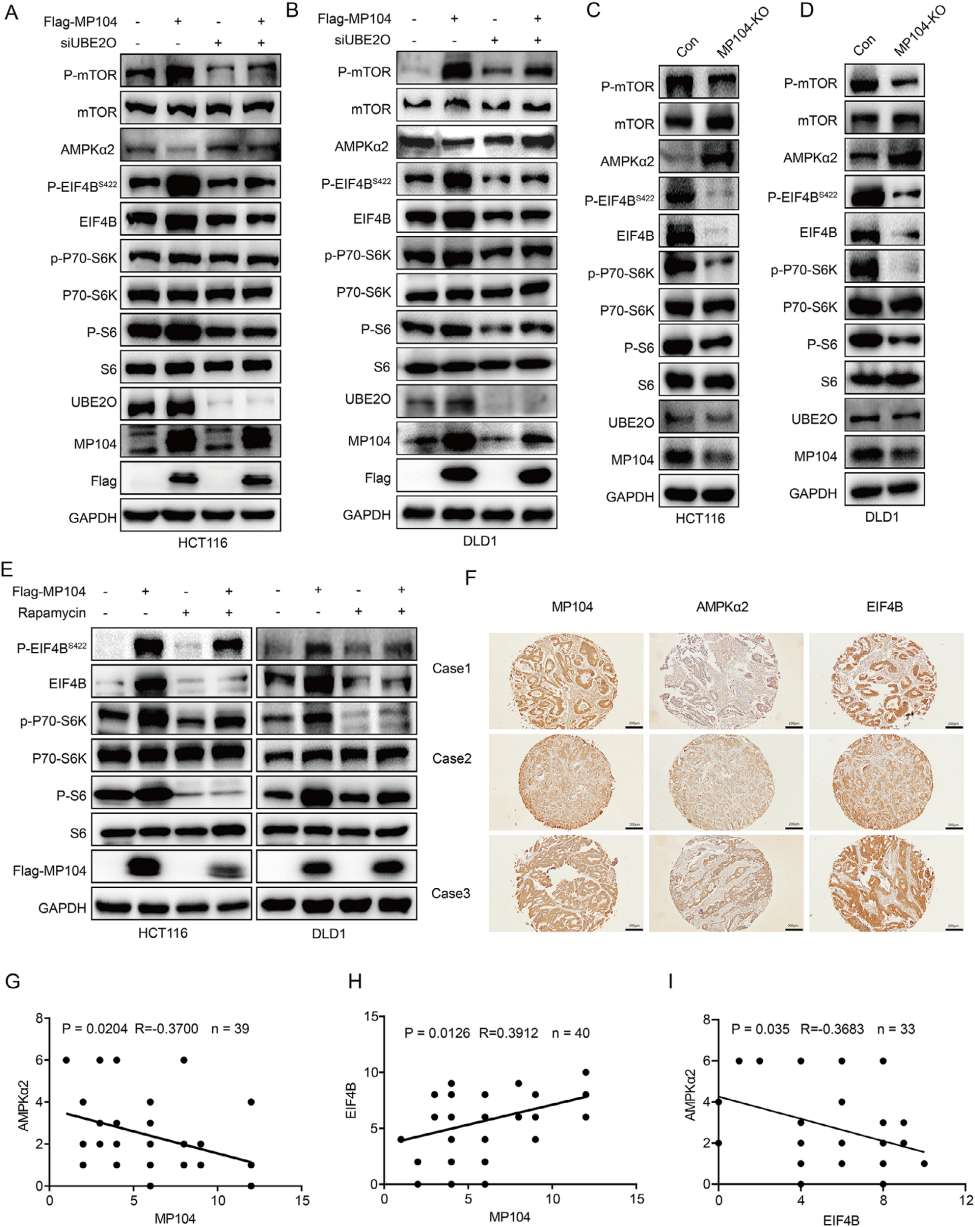
MP104 Regulates EIF4B Through the UBE2O‐AMPKα2‐mTOR Pathway. (A,B) Western blot analysis of HCT116 and DLD1 cells to assess the effects of MP104 overexpression or UBE2O knockdown (via siRNA) on the expression of AMPKα2, mTOR signaling pathway, EIF4B, and phosphorylated EIF4B, using the corresponding antibodies. (C,D) Western blot analysis of the proteins involved in the UBE2O‐AMPKα2‐mTOR‐EIF4B regulatory axis in MP104 knockout in HCT116 and DLD1 cells. (E) Lysates from transformed HCT116 cells overexpressing Flag‐MP104 and treated with rapamycin (100 nm, 24 h) were analyzed by immunoblotting. (F) Representative images of immunohistochemistry (IHC) showing the expression patterns of AMPKα2, EIF4B, and MP104 in colorectal cancer tissues. Scar bar: 200 µm. (G–I) Correlation analysis of AMPKα2, EIF4B, and MP104 expression in colorectal cancer tissues based on IHC scoring.

To determine whether these molecular relationships are reflected in clinical specimens, we performed immunohistochemical (IHC) analysis of colorectal cancer patient tissues (Figure 6F). Quantitative assessment of staining intensities revealed that across CRC samples, MP104 expression was negatively correlated with AMPKα2 levels (Figure 6G) and positively correlated with EIF4B expression (Figure 6H). Additionally, AMPKα2 expression inversely correlated with EIF4B levels (Figure 6I). These correlations in patient samples support the hypothesis that MP104 promotes AMPKα2 degradation, leading to mTOR activation and increased EIF4B phosphorylation and abundance. Together, these data demonstrate that MP104 enhances the UBE2O‐mediated degradation of AMPKα2, thereby activating mTOR signaling. Activated mTOR promotes the phosphorylation of EIF4B at Ser422 and increases EIF4B protein levels, ultimately facilitating translational upregulation in colorectal cancer cells. The MP104‐UBE2O‐AMPKα2‐mTOR‐EIF4B axis provides a mechanistic link by which MP104 regulates protein synthesis and contributes to tumor progression.

### 3.7 MP104 Inhibits RNF40‐Mediated Ubiquitination and Degradation of EIF4B

EIF4B ubiquitination and proteasomal degradation are regulated by the deubiquitinase USP11 [26], and phosphorylation at Ser422 enhances EIF4B activity [27]. Since we previously observed that MP104 increases EIF4B Ser422 phosphorylation and protein levels, we investigated whether MP104 affects EIF4B stability. First, treatment with the proteasome inhibitor MG132 blocked EIF4B degradation, confirming that EIF4B underwent proteasome‐mediated turnover (Figure 7A). Overexpression of MP104 partially abrogated EIF4B ubiquitination (Figure 7B,C). In cycloheximide (CHX) chase assays, MP104 overexpression slowed the degradation rate of EIF4B, indicating increased protein stability (Figure 7D,E). Since the E3 ubiquitin ligase responsible for EIF4B has been definitively identified, we searched the BioGRID database for candidate E3 ligases that interact with EIF4B and tested their impact on EIF4B expression. Among the candidates, only RNF40 reduced EIF4B protein levels, whereas SMURF1, Parkin, HERC5, and the deubiquitinase USP11 had no effect on EIF4B expression (Figure 7F). This result suggests that RNF40 is a likely E3 ligase for EIF4B. Co‐immunoprecipitation confirmed the physical interaction between RNF40 and EIF4B (Figure 7G,H). Ubiquitination assays further revealed that EIF4B was modified by both K11‐ and K48‐linked ubiquitin chains (Figure 7I). Next, we examined whether MP104 influenced RNF40‐mediated ubiquitination of EIF4B. MP104 overexpression inhibited the RNF40‐driven ubiquitination of EIF4B and weakened the RNF40–EIF4B interaction (Figure 7J and Figure S8). Given our earlier finding that MP104 enhances the phosphorylation of EIF4B at Ser422, and reports that Ser422 phosphorylation modulates EIF4B function, we tested whether this phosphorylation affects ubiquitination. We generated an EIF4B S422A mutant and compared its ubiquitination with that of wild‐type EIF4B in the presence of MP104. MP104 reduced ubiquitination of wild‐type EIF4B but had no effect on the S422A mutant (Figure 7K,L), indicating that MP104's inhibition of EIF4B ubiquitination depends on Ser422 phosphorylation. Together, these data support a model in which MP104 enhances the UBE2O‐AMPKα2‐mTOR axis, enhances phosphorylation of EIF4B at Ser422. Phosphorylated EIF4B is less susceptible to RNF40‐mediated ubiquitination and degradation, which leads to increased EIF4B protein levels. This mechanism contributes to the enhanced translational capacity in colorectal cancer cells.

**FIGURE 7:**
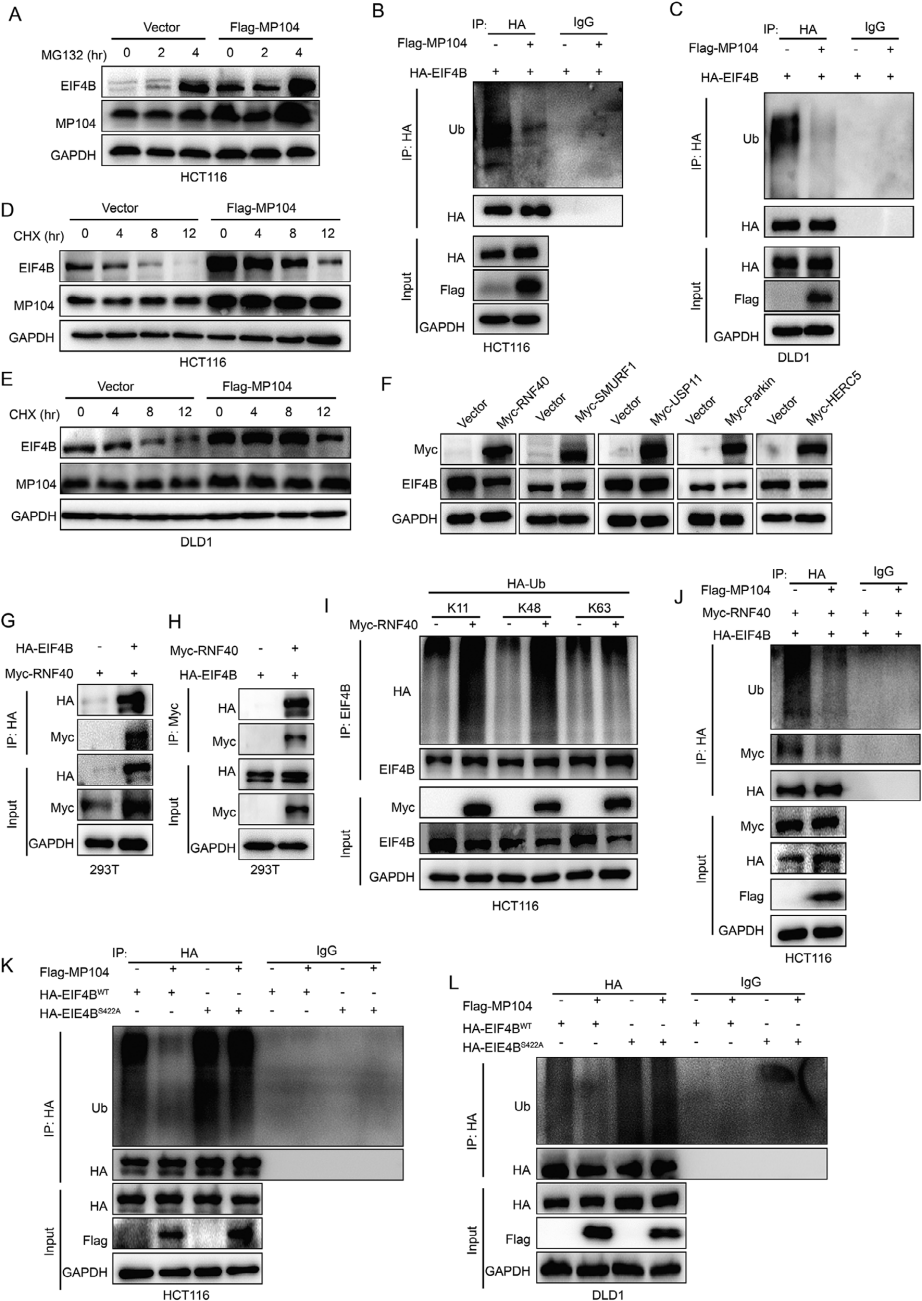
MP104 Inhibits RNF40‐Mediated Ubiquitination and Degradation of EIF4B Protein. (A) Western blot analysis showing the effects of the proteasome inhibitor MG132 (10 µm) on EIF4B protein accumulation over time in HCT116 cells expressing either vector or Flag‐MP104. (B,C) Lysates from HCT116 and DLD1 cells co‐expressing Flag‐MP104 and HA‐EIF4B were subjected to IP using anti‐HA or IgG control, followed by immunoblotting for ubiquitin to evaluate EIF4B ubiquitination. (D,E) Western blot analysis of EIF4B protein degradation in HCT116 and DLD1 cells expressing vector or Flag‐MP104 at the indicated time points after treatment with cycloheximide (CHX, 100 µg/mL). (F) Lysates from HCT116 cells expressing Myc‐tagged RNF40, SMURF1, USP11, Parkin, or HERC5 were analyzed by western blot to verify expression of the indicated proteins. (G,H) HA‐EIF4B and Myc‐RNF40 were co‐expressed in HEK293T cells, followed by IP assays to assess the interaction between EIF4B and RNF40. (I) Analysis of ubiquitin linkage specificity. HCT116 cells were transfected with plasmids expressing HA‐tagged ubiquitin mutants (K11‐only, K48‐only, or K63‐only), with or without co‐expression of Myc‐RNF40. After 48 h, cells were harvested and subjected to anti‐EIF4B immunoprecipitation and anti‐HA immunoblotting to detect ubiquitination patterns. (J) Lysates from HCT116 cells co‐expressing Myc‐RNF40 and HA‐EIF4B, with or without Flag‐MP104, were immunoprecipitated using anti‐HA or IgG control, followed by immunoblotting for ubiquitin and for the indicated proteins to assess the effect of MP104 on RNF40–EIF4B interaction and ubiquitination. (K,L) Lysates from HCT116 and DLD1 cells co‐expressing Flag‐MP104 and either wild‐type HA‐EIF4B or a phosphorylation‐deficient mutant (HA‐EIF4B S422A) were immunoprecipitated using anti‐HA or IgG control, followed by immunoblotting for ubiquitin to assess the impact of Ser422 phosphorylation on EIF4B ubiquitination.

### 3.8 MP104 Regulates CRC Malignant Progression Through the UBE2O‐AMPKα2‐mTOR‐EIF4B Pathway

To confirm whether MP104 promotes CRC malignant progression through the UBE2O‐AMPKα2‐mTOR‐EIF4B axis, we examined the role of UBE2O in MP104‐induced oncogenic functions. CCK‐8 assays showed that silencing of UBE2O significantly inhibited the proliferation of HCT116 and DLD1 colorectal cancer cells overexpressing MP104 (Figure 8A,B). Similarly, clonogenic assays demonstrated that UBE2O knockdown suppressed the clonogenic capacity of MP104‐overexpressing CRC cells (Figure 8C,D). Additionally, UBE2O knockdown inhibited the migration and invasion abilities of MP104‐overexpressing cells (Figure 8E,F), indicating that MP104 promotes CRC cell malignancy through UBE2O. Next, we explored the role of EIF4B in MP104‐regulated CRC progression. Clonogenic assays revealed that overexpression of EIF4B in MP104‐KO cancer cells significantly restored the clonogenic capacity inhibited by MP104 knockout (Figure 8G,H). Similarly, migration and invasion assays showed that EIF4B overexpression rescued the inhibitory effects of MP104 knockout on cell migration and invasion (Figure 8I,J). Finally, to assess the contribution of the mTOR pathway to MP104‐induced tumor growth in vivo, we performed subcutaneous xenograft tumor assays in nude mice using HCT116 cells overexpressing MP104. Mice were treated with rapamycin every other day. Rapamycin treatment significantly inhibited tumor growth in mice bearing MP104‐overexpressing tumors (Figure 8K,L). Consistent with the in vitro results, rapamycin treatment inhibited the mTOR pathway and reduced EIF4B phosphorylation and protein levels in the mouse model (Figure 8M). To further investigate the role of MP104 in CRC tumor growth in vivo and evaluate whether mTOR inhibition could exert additional antitumor effects under MP104 deficiency, we established subcutaneous xenograft models using MP104‐knockout (KO) cells and combined MP104 depletion with rapamycin treatment. MP104 KO alone significantly suppressed xenograft tumor growth compared with the control group. Notably, rapamycin treatment further enhanced the inhibitory effect of MP104 deficiency, resulting in an additional reduction in tumor growth (Figure S9A–C). Collectively, these data suggest that MP104 promotes malignant progression of CRC through the UBE2O‐AMPKα2‐mTOR‐EIF4B axis.

**FIGURE 8:**
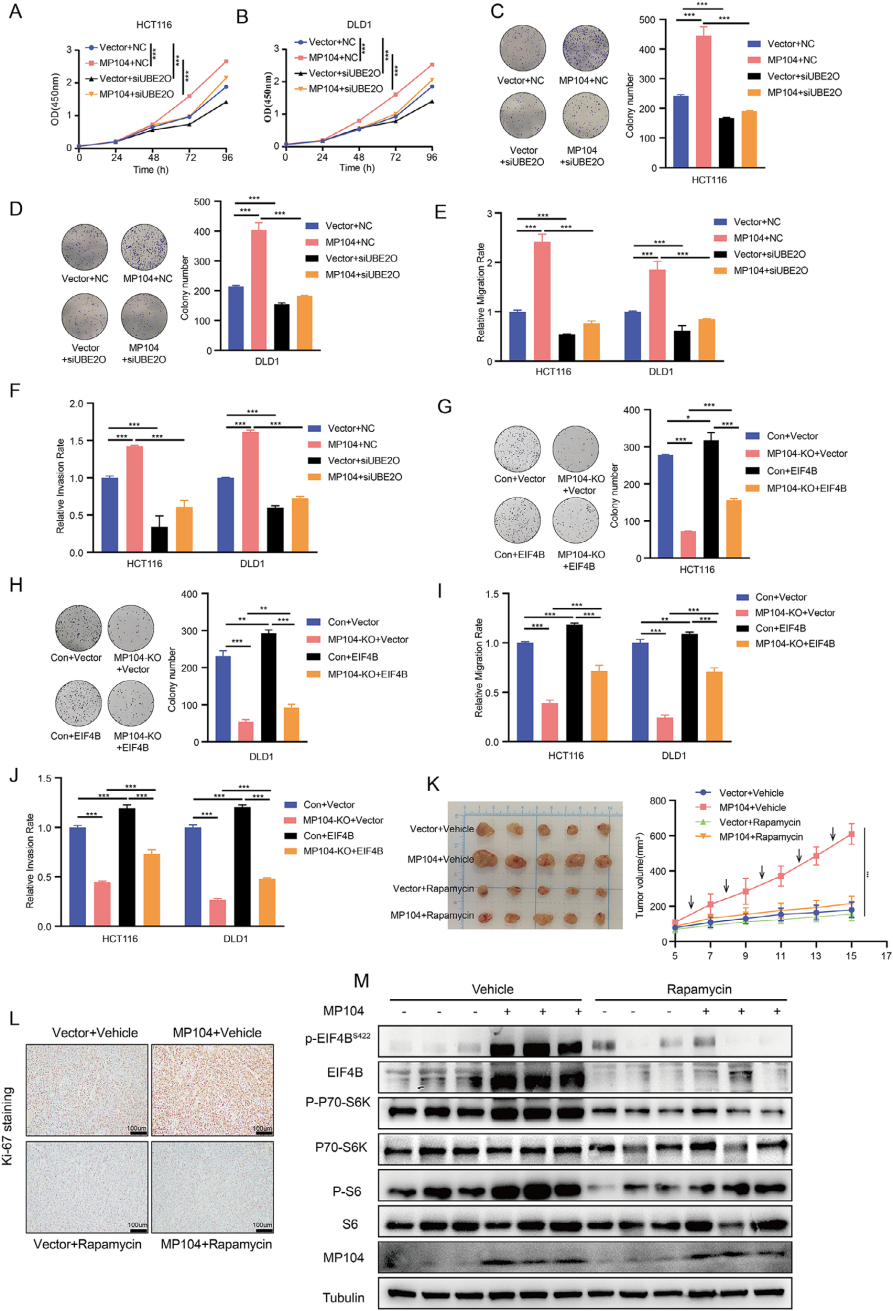
MP104 Regulates Colorectal Cancer Progression via the UBE2O–AMPKα2–mTOR–EIF4B Pathway. (A,B) CCK‐8 assays showing that UBE2O knockdown significantly suppresses the proliferation of MP104‐overexpressing HCT116 and DLD1 cells. ***p < 0.001. (C,D) Representative images and quantification of colony formation assays demonstrating that UBE2O knockdown inhibits the clonogenic capacity of MP104‐overexpressing CRC cells. ***p < 0.001. (E,F) Migration and invasion assays showing that silencing UBE2O reduces the migratory and invasive abilities of MP104‐overexpressing HCT116 and DLD1 cells. ***p < 0.001. (G,H) Colony formation assays in MP104‐knockout HCT116 and DLD1 cells with or without EIF4B overexpression, showing that EIF4B overexpression rescues the impaired clonogenic ability caused by MP104 deletion. *p < 0.05; **p < 0.01; ***p < 0.001. (I,J) Migration and invasion assays demonstrating that EIF4B overexpression reverses the inhibitory effects of MP104 knockout on CRC cell migration and invasion. **p < 0.01; ***p < 0.001. (K) In vivo xenograft tumor growth in nude mice injected with HCT116 cells overexpressing MP104 or vector control, treated with rapamycin (4 mg/kg) or vehicle. Tumor volumes were measured at the indicated time points. ***p < 0.001. (L) Representative Ki‐67 immunohistochemistry staining of xenograft tumor sections from each treatment group. Scar bar: 100 µm. (M) Western blot analysis of xenograft tumor lysates showing relative protein expressions under indicated treatment or conditions.

## 4 Discussion

Recent discoveries have challenged the long‐held view that lncRNAs lack coding potential, revealing that many lncRNAs harbor smORFs capable of encoding functional microproteins with regulatory roles in cancer and other diseases [28, 29]. In this study, we identified a previously uncharacterized microprotein, MP104, encoded by ZEB1‐AS1, and demonstrated its critical role in driving CRC progression. While ZEB1‐AS1 has been associated with tumorigenesis in multiple cancers and functions as a non‐coding RNA [19], our study is the first to highlight its protein‐coding capacity as the primary driver of its oncogenic effects in CRC. MP104 adds to this emerging class of regulatory molecules and exemplifies the importance of investigating lncRNA translation to uncover the hidden layers of cancer regulation.

We found that MP104 was significantly overexpressed in CRC tissues and cell lines compared to normal controls, and its high expression correlated with poor prognosis, suggesting its potential as a prognostic biomarker. Functional experiments confirmed that MP104 promoted CRC cell proliferation, migration, and invasion in vitro, and enhanced tumor growth and metastasis in vivo. These findings are consistent with previous reports that microproteins encoded by lncRNAs, such as HOXB‐AS3, LINC00665, LINC00493, and AC115619, can modulate tumor behavior by interacting with key regulatory proteins or complexes [10, 11, 30, 31].

EIF4B enhances the helicase activity of EIF4A to facilitate ribosome scanning through structured 5′‐UTRs, thereby promoting efficient cap‐dependent translation initiation, particularly for mRNAs encoding oncogenic proteins [32, 33]. EIF4B activity is regulated by post‐translational modifications: phosphorylation at Ser422 and Ser406, primarily by S6K1 or MELK, enhances its pro‐translation capacity [34, 35]. In this study, we found that MP104 binds to the E2/E3 ubiquitin ligase UBE2O, enhancing its interaction with and ubiquitination of AMPKα2, a central energy sensor and negative regulator of the mTOR pathway [15]. The resulting degradation of AMPKα2 leads to activation of mTOR signaling and increased phosphorylation of EIF4B. Moreover, our study demonstrated that MP104 enhances EIF4B phosphorylation and stability by inhibiting RNF40‐mediated ubiquitination and degradation. This finding is particularly significant given the limited understanding of the post‐translational modifications that regulate EIF4B. Although previous studies have identified EIF4B as a substrate for ubiquitination and degradation, the specific E3 ubiquitin ligases and regulatory mechanisms involved remain largely unknown. Our identification of RNF40 as a potential E3 ubiquitin ligase for EIF4B and the role of MP104 in modulating this process provides new avenues for research into the regulation of protein translation in cancer cells.

Previous studies have shown that lncRNA‐encoded microproteins exert their biological functions through diverse molecular mechanisms. For example, the microprotein ASAP encoded by LINC00467 regulates CRC progression through modulation of mitochondrial ATP synthase activity [9], whereas the microprotein CIP2A‐BP encoded by LINC00665 suppresses breast cancer metastasis by competing with PP2A for CIP2A binding, thereby inhibiting oncogenic PI3K/AKT/NF‐κB signaling [10]. Like these reported microproteins, our findings suggest that MP104 also exerts its function through interactions with binding partners. However, unlike previously characterized mechanisms, MP104 promotes the interaction between UBE2O and AMPKα2, thereby facilitating UBE2O‐mediated degradation of AMPKα2. These findings suggest that microproteins may play important roles in regulating protein complex assembly, enzymatic activity, and ubiquitin‐dependent protein turnover. Collectively, our results further support the concept that lncRNA‐derived microproteins can exert biological functions through distinct and diverse regulatory mechanisms.

Importantly, the mTOR‐EIF4B axis has been previously implicated in cancer cell survival, proliferation, and stress resistance [26, 36]. Our results suggested that MP104 operates as a critical upstream regulator of this axis, coordinating both ubiquitin‐mediated protein degradation and translational control. The pharmacologic inhibition of mTOR by rapamycin effectively suppressed MP104‐driven tumor growth in vivo, underscoring the therapeutic relevance of this pathway and highlighting MP104 as a potential target for mTOR‐directed therapies in CRC. The clinical relevance of our findings is underscored by the strong correlation between MP104 expression and poor prognosis in CRC patients. The upregulation of MP104 in CRC tissues and its association with advanced tumor stages and reduced survival highlight its potential as a biomarker for disease progression and therapeutic targeting. Given the central role of the UBE2O‐AMPKα2‐mTOR‐EIF4B axis in cancer biology, targeting this pathway may offer new therapeutic opportunities for CRC treatment. For instance, inhibitors of UBE2O or activators of AMPKα2 can potentially disrupt the oncogenic effects of MP104, providing a novel strategy for cancer treatment.

Despite the significance of our findings, several limitations of the current study should be acknowledged. Although we established MP104 as a critical regulator of CRC progression through the UBE2O–AMPKα2–mTOR–EIF4B axis, the precise structural and molecular determinants governing MP104‐mediated regulation remain to be fully elucidated. Given the emerging recognition that microproteins often function as modulators of protein complexes, it will be important to define how MP104 dynamically influences protein interactions and ubiquitin signaling at a broader systems level. In addition, while our in vitro and in vivo data support the oncogenic role of MP104, further validation in larger patient cohorts and clinically relevant models will be necessary to establish its translational significance. Future studies integrating structural biology, proteomics, and clinical investigations may provide a more comprehensive understanding of the biological functions and therapeutic potential of MP104 in colorectal cancer.

## 5 Conclusions

In conclusion, our study provides a comprehensive understanding of the role of MP104 in CRC progression, highlighting its innovative aspects in the context of lncRNA‐encoded microproteins and reveling the MP104‐UBE2O‐AMPKα2‐mTOR‐EIF4B signaling axis. These findings reveal a previously unknown coding function of ZEB1‐AS1 and propose MP104 as a promising diagnostic marker and therapeutic target in colorectal cancer.

## Author Contributions

**Fang Chen**: investigation, formal analysis, data curation, software, validation, conceptualization, methodology. **Qirui Ge**: investigation. **Benli Li**: investigation. **Miao Wang**: investigation, methodology, formal analysis, data curation, validation. **Lei Zhang**: investigation. **Li Lin**: investigation. **Qianqian Xiao**: investigation. **Sufang Chu**: investigation. **Jin Ding**: investigation, formal analysis. **Jin Bai**: writing – original draft, writing – review and editing, funding acquisition, project administration, supervision, software. **Pingfu Hou**: writing – original draft, writing – review and editing, funding acquisition, visualization, project administration, supervision. **Qianqing Wang**: writing – review and editing. **Shiping Xu**: investigation, formal analysis, resources. **Hongmei Yong**: investigation, formal analysis. **Yan Wang**: investigation. All the authors approved the final version of the manuscript .

## Ethics Approval and Consent to Participate

This study was conducted in compliance with the principles of the Declaration of Helsinki. Informed consent was obtained from all subjects. Ethical approval for the study was obtained from the Ethics Committee of the Affiliated Hospital of Xuzhou Medical University. Ethical approval for animal experiments was provided by the Institutional Animal Care and Use Committee of Xuzhou Medical University.

## Conflicts of Interest

The authors declare no conflicts of interest.

## Supporting information




**Supporting File 1**: advs76593‐sup‐0001‐SuppMat.docx.

**Supporting File 2**: advs76593‐sup‐0002‐Table S1.xlsx.

## Data Availability

Data sharing not applicable to this article as no datasets were generated or analyzed during the current study.
