# Supplementary material for: Microprotein MP104 Promotes Malignant Progression of Colorectal Cancer Through Regulating Protein Translation
Source: Adv Sci (Weinh). 2026 Jul 23:e76593. Online ahead of print. doi: 10.1002/advs.76593 (PMC13393260; doi:10.1002/advs.76593)
Supplement: Supplementary file 1 — Supporting File 1: advs76593‐sup‐0001‐SuppMat.docx. [file ADVS-9999-e76593-s002.docx]

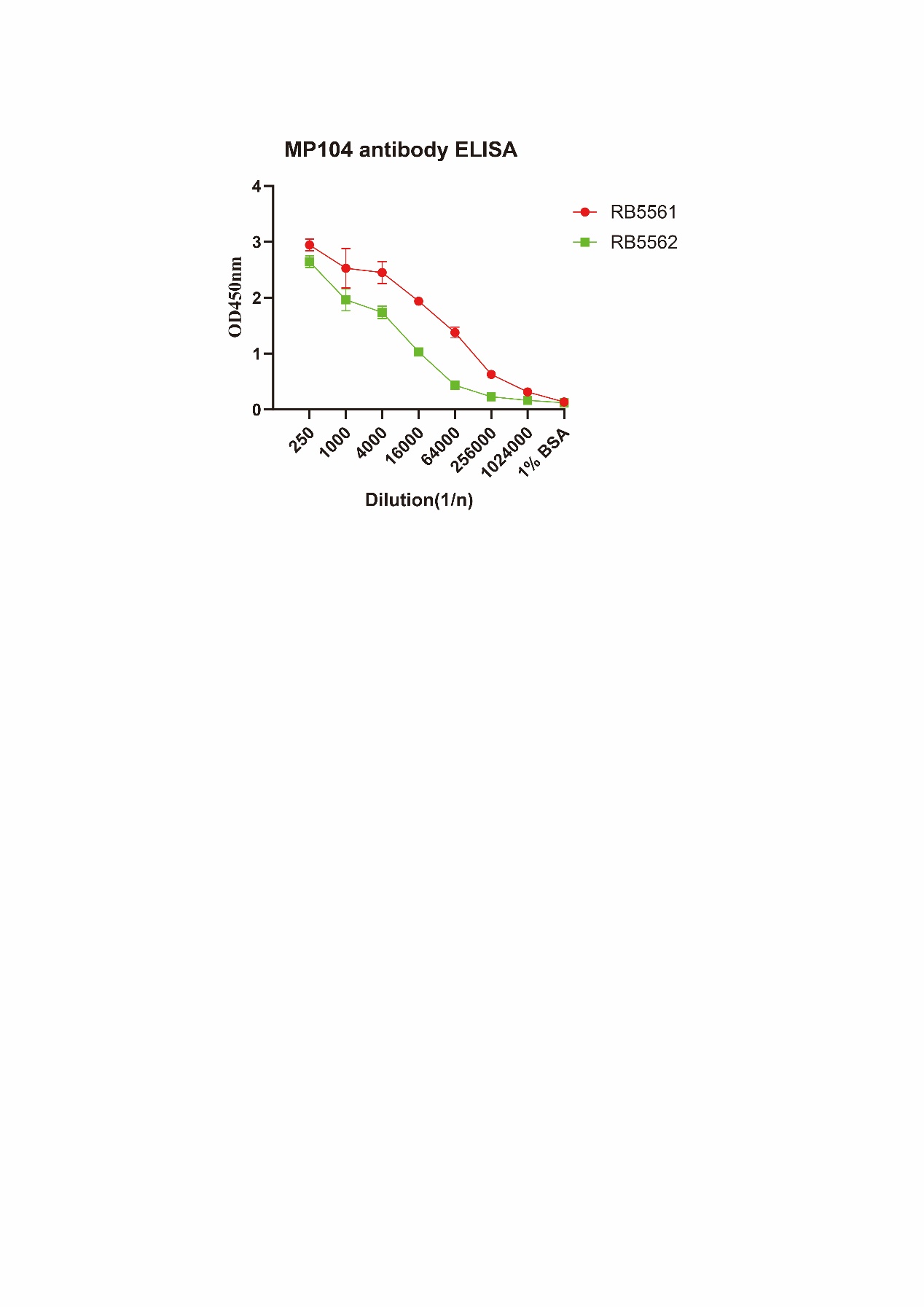


**Figure S1**. **ELISA assays for custom-made antibodies against MP104.** Two antibodies (RB5561 and RB5562) against MP104 were used to test the sensitivity of the antibodies. The dilutions of the relative antibodies were showed as listed, two replicated tests were performed.

**
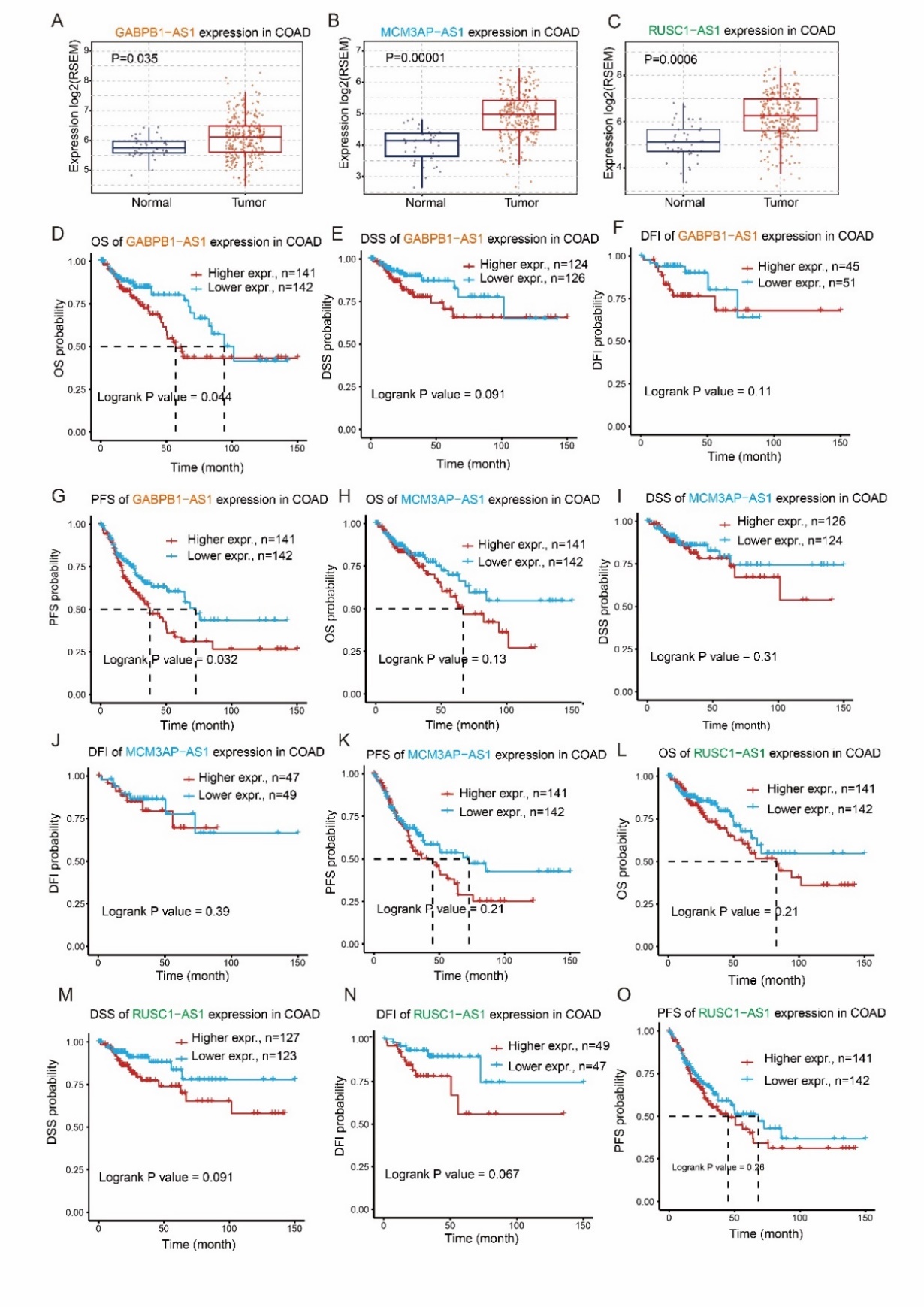
Figure S2. Relative expression and survival analysis of MCM3AP-AS1, RUSC1-AS1 and GABPB1-AS1 in CRC.** (A-C) Relative expressions of MCM3AP-AS1, RUSC1-AS1 and GABPB1-AS1 in CRC against normal tissues. (D-G) Survival analysis of GABPB1-AS1 in CRC. (H-K) Survival analysis of MCM3AP-AS1 in CRC. (L-O) Survival analysis of RUSC1-AS1 in CRC. Relative data was analyzed in GSCA database.


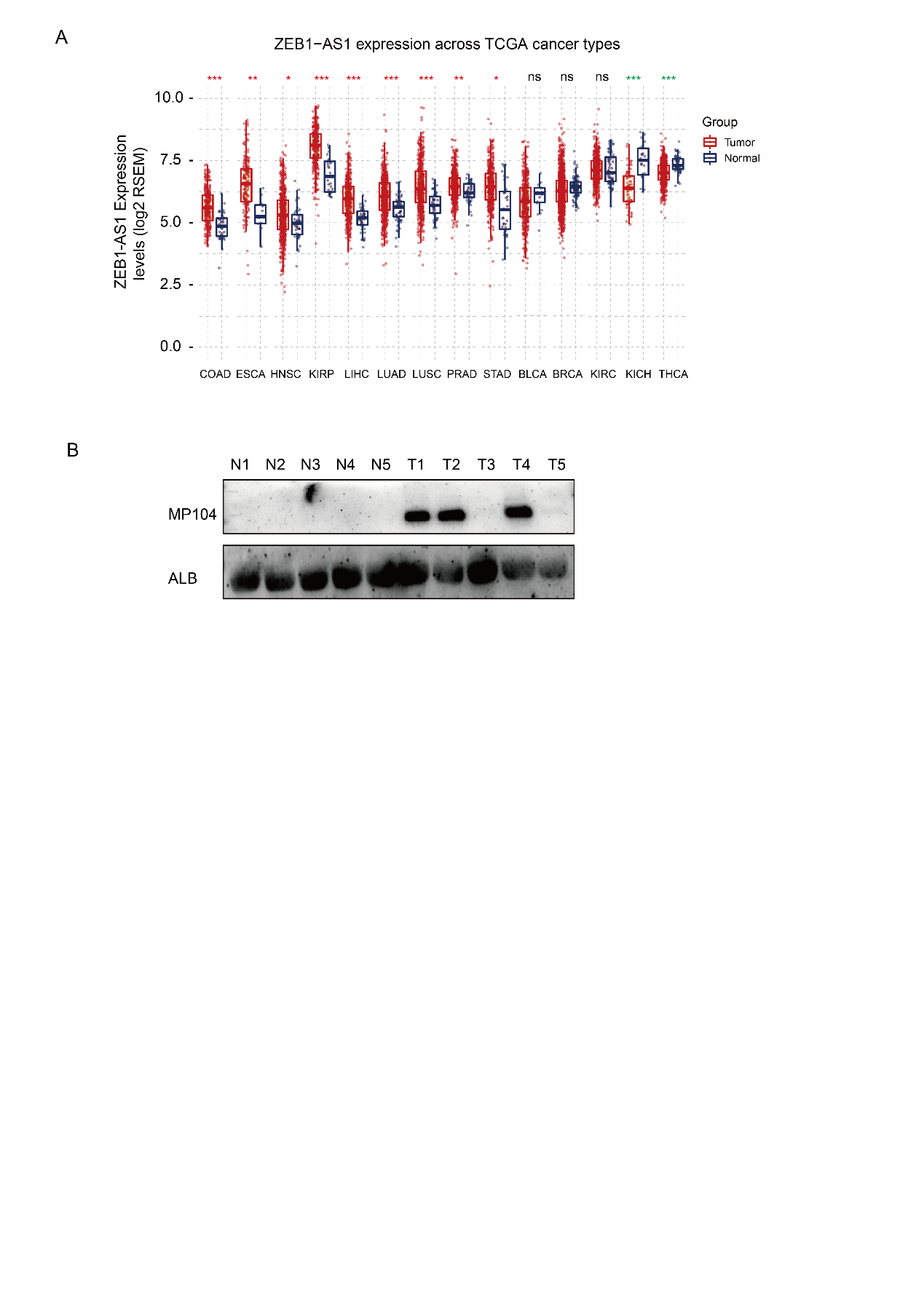


**Figure S3. ZEB1−AS1 expression across TCGA cancer types and detection of MP104 in Serum. (A)** GSCA database analysis revealed that ZEB1-AS1 is significantly upregulated in 9 types of cancers and downregulated in 2 cancer types. *P<0.05, **P<0.01, ***P<0.001. (B) Detection of MP104 by Western blot, Albumin (ALB) served as a loading control for normalization.


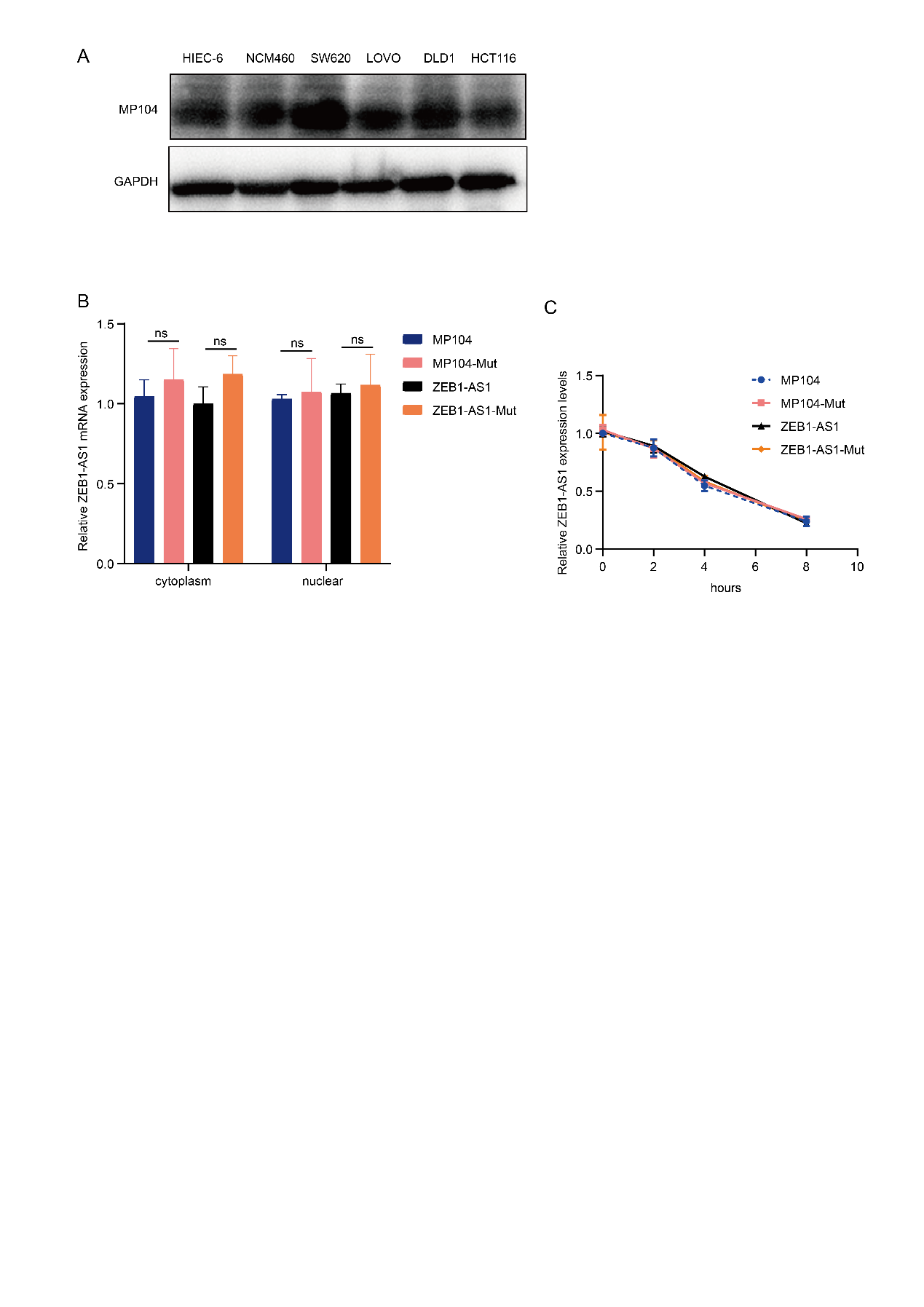


**Figure S4. Expression of MP104 in different cell lines.** **(A)** Relative expression of MP104 protein levels analyzed by Western blotting. GAPDH was used as loading control. **(B)** Nuclear and cytoplasmic fractionation followed by qRT-PCR analysis was performed to determine the expression levels of ZEB1-AS1 mRNA among the MP104, MP104-Mut, ZEB1-AS1, and ZEB1-AS1-Mut groups. **(C)** RNA stability assays were performed using Actinomycin D (10 ug/ml) treatment. Comparable RNA decay rates were observed among the four groups, ZEB1-AS1 levels at different time points were detected by q-RT-PCR, RNA levels were normalized to GAPDH.


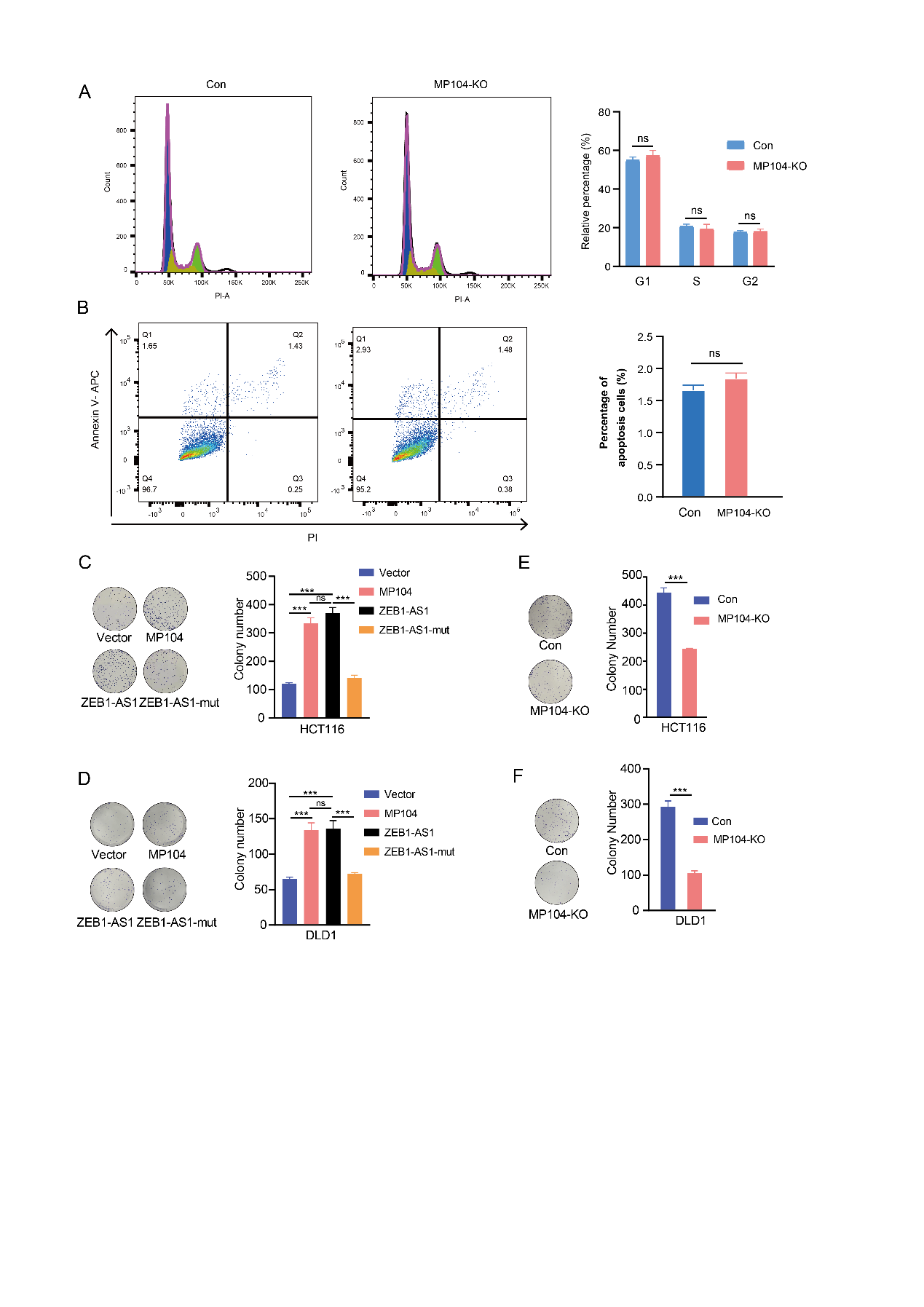


**Figure S5. Effects of MP104 on cell cycle progression, apoptosis, and colony formation.** **(A)**Cell cycle distribution of MP104-knockout HCT116 cells was analyzed by flow cytometry. **(B)** Apoptosis of MP104-knockout HCT116 cells was analyzed by flow cytometry. **(C-F)** Colony formation assays were conducted to evaluate the proliferative capacity of the corresponding cell lines. Data are presented as mean ± SD from three biologically independent experiments. Two-way ANOVA was used for statistical analysis. ***P < 0.001.

**
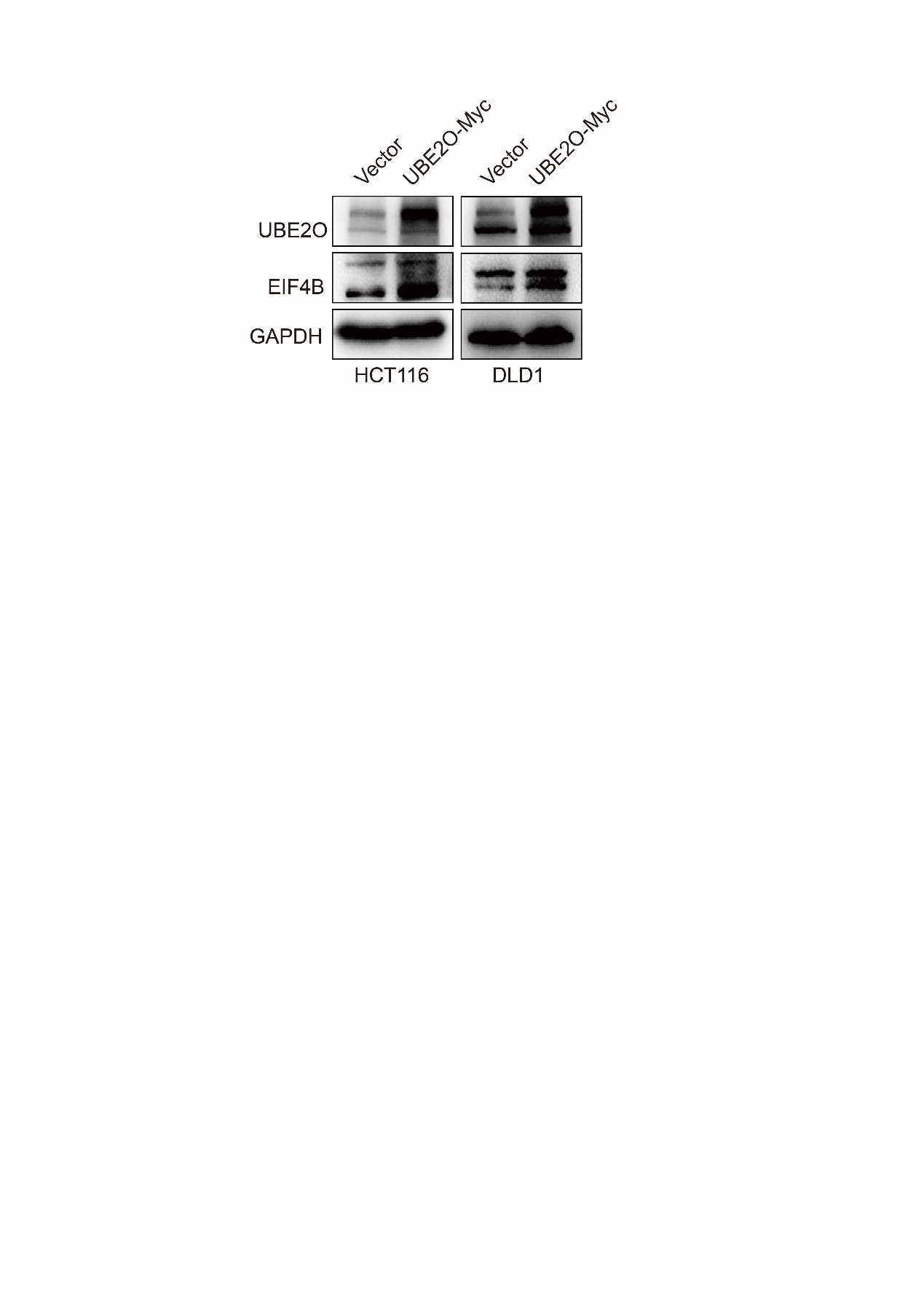
**

**Figure S6. UBE2O promotes EIF4B protein expression in CRC.** EIF4B protein levels were upregulated in UBE2O overexpressed HCT116 and DLD1 cells. GAPDH was used as loading control.


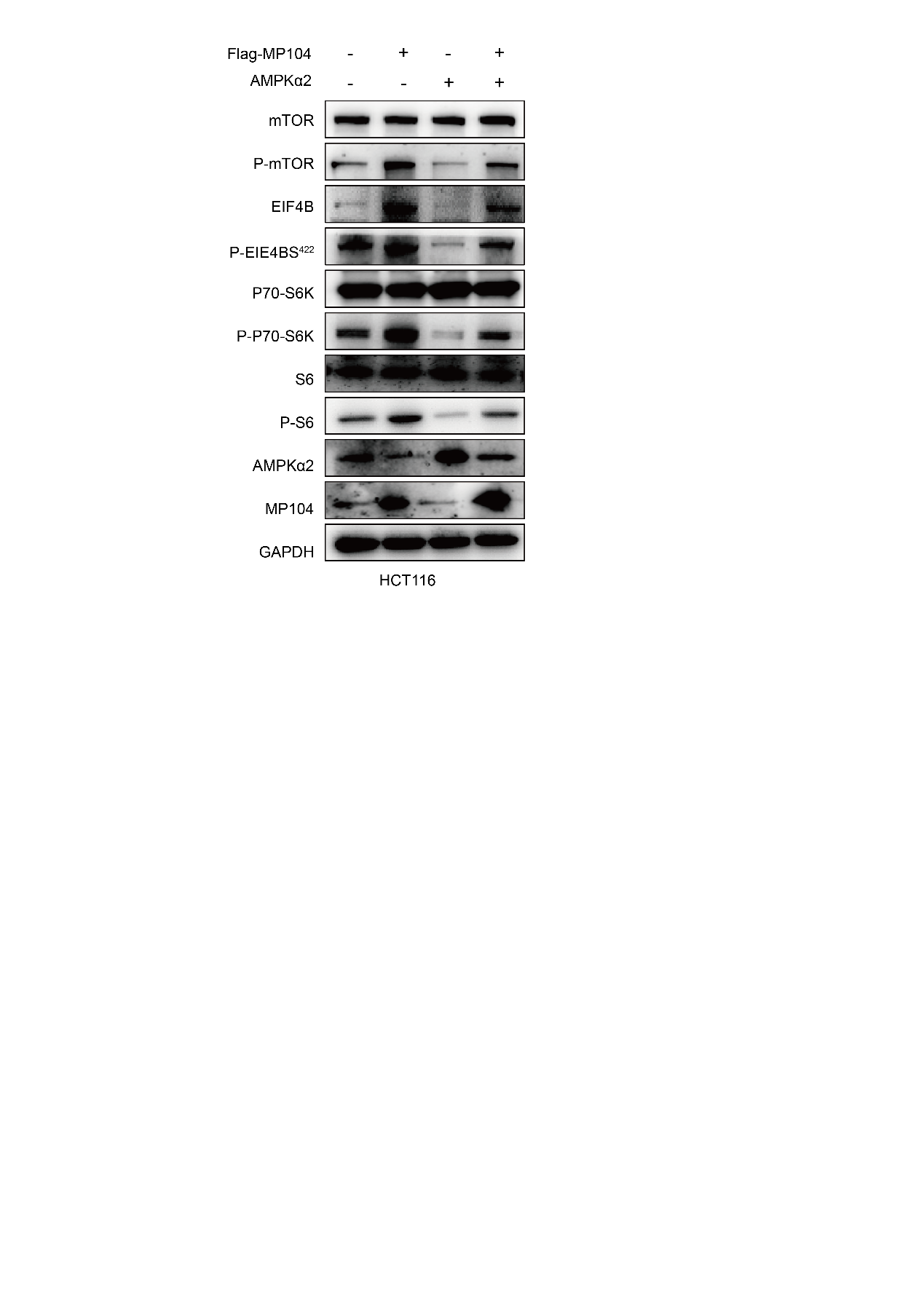


**Figure S7. Overexpression of AMPKα2 rescued MP104-induced activation of the mTOR signaling pathway**. Western blot analysis showed Co-overexpression of AMPKα2 attenuated MP104-induced activation of the mTOR signaling pathway, as indicated by reduced phosphorylation of mTOR, P70-S6K, S6, and EIF4B

**
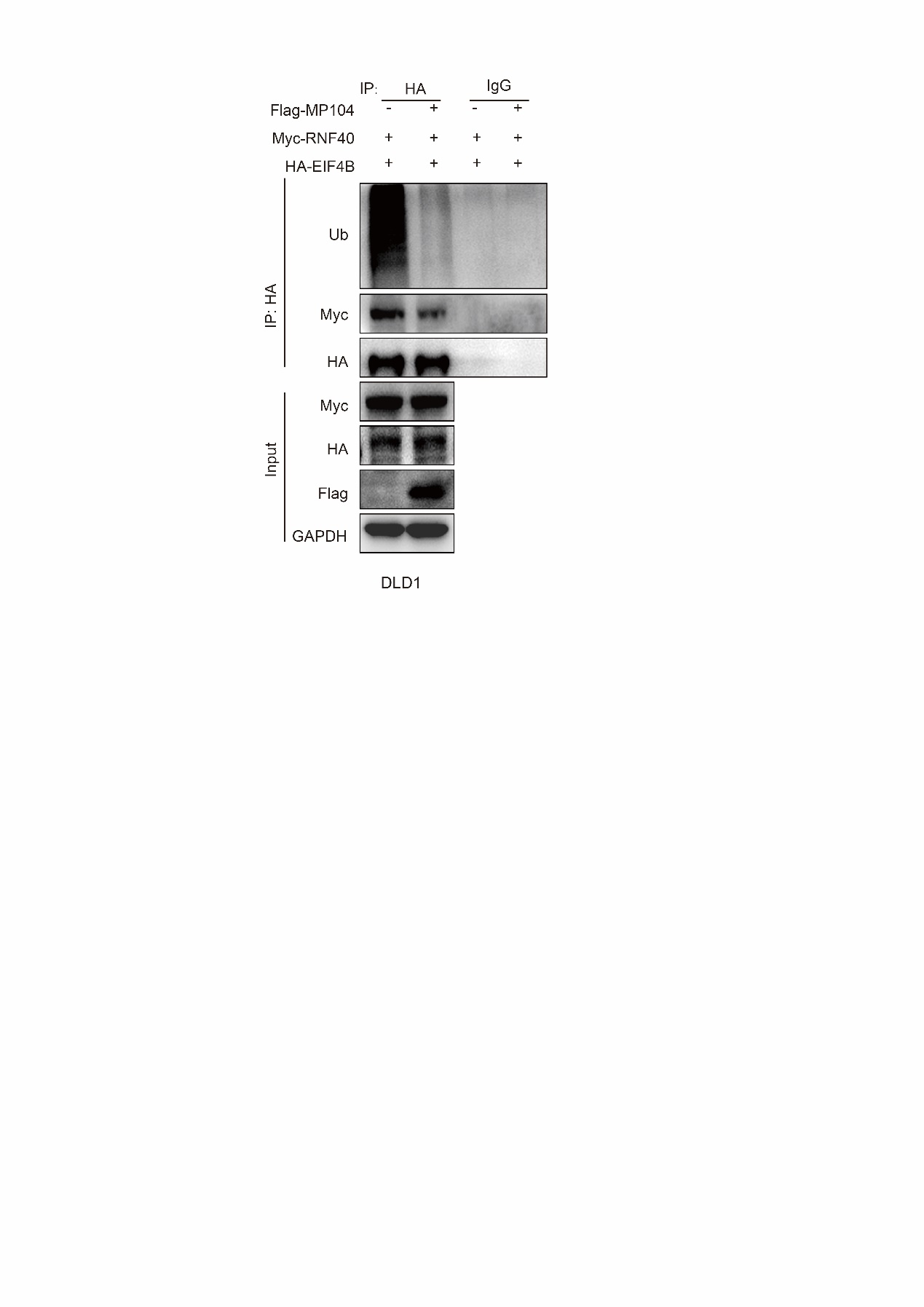
****Figure S8. MP104 Inhibits RNF40-Mediated Ubiquitination of EIF4B Protein.** Lysates from DLD1 cells co-expressing Myc-RNF40 and HA-EIF4B, with or without Flag-MP104, were immunoprecipitated using anti-HA or IgG control, followed by immunoblotting for ubiquitin and for the indicated proteins to assess the effect of MP104 on RNF40–EIF4B interaction and ubiquitination.

**
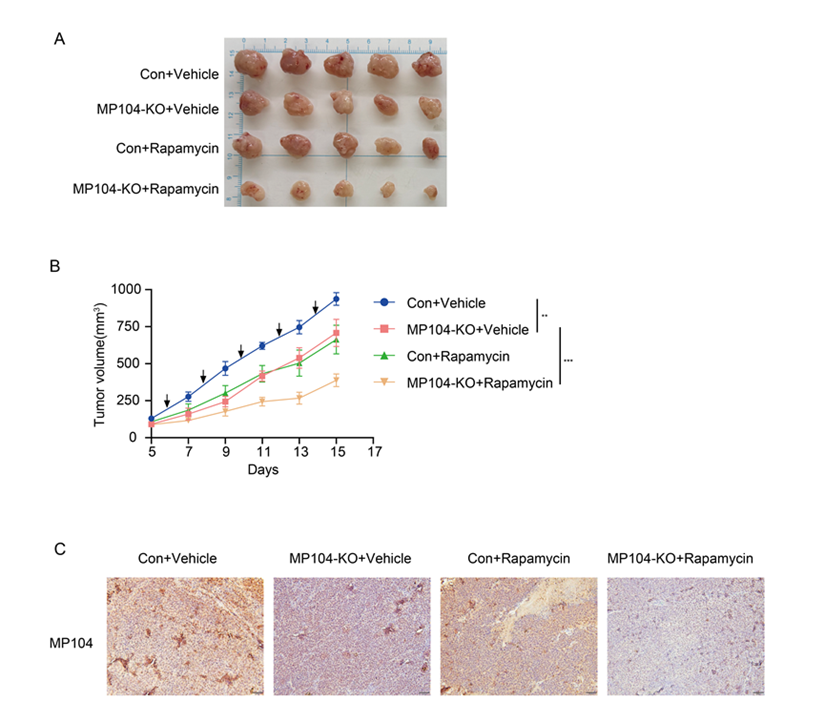
**

**Figure S9. MP104 knockout repressed CRC tumor growth.** (A-B) In vivo xenograft tumor growth in nude mice injected with HCT116 cells MP104 knockout or vector control, treated with rapamycin or vehicle. Tumor volumes were measured at the indicated time points. **P <0.01. ***P < 0.001. (C) Representative MP104 immunohistochemistry staining of xenograft tumor sections from each treatment group. Scar bar: 50 μm.

**Supplementary materials and methods**

**Cell Cultures and Treatment**

The cell lines (HCT116, DLD-1, 293T) were obtained from the Cell Bank of the Chinese Academy of Sciences. Cells were cultured in DMEM supplemented with 10% FBS and 1% penicillin-streptomycin in a 5% CO2 atmosphere at 37 °C. Plasmid transfection was performed using jetPRIME® (101000046, Polyplus) or polyethyleneimine (PEI, 23966-1, Polysciences) according to the manufacturer's instructions.

**Co-Immunoprecipitation**

Cells were cultured in 10 cm dishes until reaching 80-90% confluency. Cells were harvested and lysed in 1 mL of IP lysis buffer (P0013J, Beyotime) supplemented with a complete protease inhibitor cocktail. Lysis was performed on ice for 30 minutes with mixing every 5 minutes. The lysates were centrifuged at 12,000 rpm for 10 minutes at 4°C, and the supernatant was collected. For immunoprecipitation, 5 µg of antibody was incubated with Protein A/G magnetic beads at 4°C for 1 hour with gentle rotation. The antibody-bead complex or anti-FLAG Magnetic Beads, anti-HA magnetic beads, or anti-c-Myc Magnetic Beads and respective controls were then incubated with the collected cell lysate overnight at 4°C. After incubation, the beads were washed three times with lysis buffer to remove nonspecifically bound proteins. An appropriate amount of SDS buffer was added to the beads, which were then heated at 95°C for 5 minutes. The proteins were separated by SDS-PAGE electrophoresis and detected by western blot.

**Immunofluorescence Staining and Confocal Microscopy**

Cells were seeded on glass coverslips and cultured under standard conditions. For immunofluorescence staining, cells were fixed with 4% paraformaldehyde (PFA) in phosphate-buffered saline (PBS) for 15 minutes at room temperature, followed by permeabilization with 0.1% Triton X-100 in PBS for 10 minutes. After washing with PBS, cells were blocked with 5% bovine serum albumin (BSA) in PBS for 1 hour at room temperature to prevent nonspecific antibody binding. Subsequently, cells were incubated with the primary antibody (anti-Flag, 1:500) diluted in 1% BSA in PBS at 4°C overnight. The next day, cells were washed three times with PBS and incubated with the appropriate fluorophore-conjugated secondary antibody for 1 hour at room temperature in the dark. Nuclei were counterstained with DAPI (1 µg/mL) for 5 minutes and then washed three times with PBS. Finally, coverslips were mounted using anti-fade mounting medium and imaged using a fluorescence microscope (ZEISS LSM880, Germany).

**Treatment with MG132, Cycloheximide, Rapamycin or actinomycin D**

For protein stability and degradation assays, HCT116 and DLD1 cells were cultured in 60 mm dishes until reaching 80-90% confluency. Cells were then treated with MG132 (10 µM) for indicated time points to inhibit proteasomal degradation. For protein half-life analysis, cells were treated with cycloheximide (CHX, 100 µg/mL) to block protein synthesis and harvested at the indicated time points for subsequent analysis. For rapamycin treatment, HCT116 cells were treated with rapamycin (100 nM) for 24 hours and then harvested for immunoblotting. RNA stability was measured by incubating cells with 10 mg/ml actinomycin D (Act. D) and calculating the relative ZEB1-AS1 mRNA levels at different time points using q-RT-PCR. RNA levels were normalized to GAPDH.

**Ubiquitination assays**

To assess the ubiquitination status of EIF4B, ubiquitin mutant constructs K11, K48, and K63 were generated. In each construct, all lysine (K) residues of ubiquitin were mutated to arginine (R) except for lysine 11 (K11), lysine 48 (K48), or lysine 63 (K63), respectively, thereby allowing the formation of only K11-, K48-, or K63-linked polyubiquitin chains. HA-tagged K11-, K48-, or K63-only ubiquitin plasmids were co-transfected with Myc-RNF40 into HCT116 cells. Forty-eight hours after transfection, immunoprecipitation was performed using an anti-EIF4B antibody. The ubiquitination status and linkage pattern of EIF4B were then analyzed by immunoblotting with an anti-HA antibody.

Flow cytometry

For cell cycle analysis, cells were stained using the Cell Cycle Detection Kit (KGA9101-100, KeyGEN BioTECH) according to the manufacturer’s instructions and analyzed using a BD LSRFortessa flow cytometer. For apoptosis analysis, cells were stained using the Annexin V-APC/PI Apoptosis Detection Kit (KGA1107-50, KeyGEN BioTECH) according to the manufacturer’s protocol and subsequently analyzed using a BD FACSCanto II flow cytometer. Data were analyzed using FlowJo software.

**Real-time qPCR**

Total RNAs were extracted with the RNA isolater reagent (R401-01-AA, Vazyme) and reverse-transcribed with the PrimeScript First Strand cDNA Synthesis kit ((RT101-01, Vazyme Biotech, Nanjing, China,). Real-time PCR was performed on an LightCycler480 II instrument using the UltraSYBR One Step RT-qPCR Kit (CW3008, CWbio, Beijing, China). Primers for RT-qPCR are: U6: 5’-CTCGCTTCGGCAGCACA-3’, 5’-AACGCTTCACGAATTTGCGT-3’; GAPDH: 5’-AAGGTCGGAGTCAACGGATTTG-3’, 5’-CCATGGGTGGAATCATATTGGAA-3’; ZEB1-AS1: 5’-GGGATCTGGCTGATTCTCCC-3’, 5’-GTGGGACTGATGGTAGCCCT-3’.

**Serum proteins extraction and detection**

Serum proteins were concentrated and extracted using a Trichloroacetic Acid Protein Concentration Kit (KGB1101-50, KeyGEN BioTECH) according to the manufacturer’s protocol. Equal amounts of extracted proteins were subjected to Western blot analysis to evaluate MP104 expression. Albumin (ALB) served as a loading control for normalization.

**Polysome Profiling**

Polysome profiling assay was performed as previously described with slight modifications^[^[^1^](#_ENREF_1)^]^. Briefly, control or MP104-overexpressing HCT116 cells were seeded in 10 cm dishes and cultured for 24 hours, followed by serum starvation for 16 hours and then re-stimulation with fresh complete medium for 2 hours. Cells were treated with cycloheximide (100 µg/mL) for 2 minutes at 37°C to stabilize ribosome-mRNA complexes. After treatment, cells were washed with ice-cold PBS, scraped, pelleted, and lysed in RIPA buffer supplemented with RNase and protease inhibitors. Lysates were clarified by centrifugation at 13,000 rpm for 10 minutes at 4°C. Supernatants were loaded onto 10-50% sucrose gradients prepared in polysome buffer (15 mM Tris-HCl, pH 7.4, 15 mM MgCl₂, 150 mM NaCl) and centrifuged at 35,000 rpm for 2 hours at 4°C using an SW41Ti rotor. Gradients were fractionated while continuously monitoring absorbance at 260 nm, and polysome profiles were generated accordingly.

**Translation Assays**

For SUnSET assays^[^[^2^](#_ENREF_2)^]^, HCT116 or HEK293T cells were seeded in 60 mm dishes and transfected with either an empty vector or pCDH-CMV-MP104-MCS-EF1-blast. Forty-eight hours post-transfection, cells were either subjected to overnight serum starvation or left untreated. For serum stimulation experiments, cells were incubated in complete medium containing 10% FBS for 2 hours following serum starvation. Subsequently, cells were treated with puromycin (20 µg/mL) for 4 hours to label nascent proteins. After treatment, cells were washed with cold PBS and lysed in RIPA buffer supplemented with 1 mM PMSF and a protease inhibitor cocktail. The whole-cell lysate was analyzed by western blot using an anti-puromycin antibody to detect protein synthesis. For SUnSET assays in MP104 knockout cells, wild-type or MP104-KO HCT116 cells were seeded in 60 mm dishes. After 24 hours, cells were transfected with either an empty vector or the pLV3-CMV-EIF4B-3×HA-Blast plasmid. Forty-eight hours post-transfection, cells were treated with puromycin (20 µg/mL) for 4 hours, followed by lysis and detection as described above.

**Immunohistochemistry (IHC)**

CRC tissues and matched adjacent normal colorectal tissues were harvested from patients at the Affiliated Hospital of Xuzhou Medical University. The use of these specimens for research purposes was granted approval by the Ethics Committee of the Affiliated Hospital of Xuzhou Medical University. IHC assays were performed as follows: Paraffin-embedded tissue sections were deparaffinized in xylene and rehydrated through a graded ethanol series. Antigen retrieval was performed by heating the sections in citrate buffer using a microwave. Endogenous peroxidase activity was blocked by treatment with 3% hydrogen peroxide for 10 minutes. The sections were then blocked with 5% normal serum at room temperature for 30 minutes and incubated overnight at 4°C with primary antibodies against MP104, AMPKα2, EIF4B, and Ki67, respectively. After washing, the sections were incubated with HRP-conjugated secondary antibodies at room temperature for 30-60 minutes. Signal detection was performed using a DAB substrate, followed by counterstaining with hematoxylin. Finally, the sections were dehydrated, cleared, and mounted for microscopic analysis.

**MP104 sequence**

ORF Sequence:

ATGCCGGGAAACCGTAGGGACGCGGTCAGAAAGGCGACGGGCTGTCGGAGTTGGAAAGGTAAAGTTGGAGGCTCGGCGGCGTCCTGCCTCCGCGCTGGCCACACCCGCCGCGGCTGCCCGGGGCAGGGAGGGATCTGGCTGATTCTCCCTGTACCCTGTGCCCTCGGAGCTGCCCCTCCGAGCGCCCAGAGGTCCCCGCCTGCCTGCTTCCTGGAGGCAGGGCTACCATCAGTCCCACGCCTCGCGTGTCCGCCCCCCGCACCCCGGGGCCAAGGAAAGGGATCGCGGTCTGGACTCCCCGGGGAGTTCCCT

MP104 Protein Sequence:

MPGNRRDAVRKATGCRSWKGKVGGSAASCLRAGHTRRGCPGQGGIWLILPVPCALGAAPPSAQRSPPACFLEAGLPSVPRLACPPPAPRGQGKGSRSGLPGEFP

**Donor DNA Sequence**

ACGACACTCCCGGCTTTACGACATCACCTTCCTTACACCTAGAGGCTCTCGCTCTACGGCCGGAACCTTGTTGCTAGGGACCGGGCGGTTTGCGGCAACCGTGGGCACTGCTGAATTTGAATTGAGGGGCGAGGGAAAAGTTTTCCTCAGGTGTGGTGGGGAGAGGGAGGCGGATGCCGGGAAACCGTAGGGACGCGGTCAGAAAGGCGACGGGCTGTCGGAGTTGGAAAGGTAAAGTTGGAGGCTCGGCGGCGTCCTGCCTCCGCGCTGGCCACACCCGCCGCGGCTGCCCGGGGCAGGGAGGGATCTGGCTGATTCTCCCTGTACCCTGTGCCCTCGGAGCTGCCCCTCCGAGCGCCCAGAGGTCCCCGCCTGCCTGCTTCCTGGAGGCAGGGCTACCATCAGTCCCACGCCTCGCGTGTCCGCCCCCCGCACCCCGGGGCCAAGGAAAGGGATCGCGGTCTGGACTCCCCGGGGAGTTCCCTGACTACAAGGACCACGACGGTGACTACAAGGACCACGACATCGACTACAAGGACGACGACGACAAGTGATGAGGGGAAGGGCAGGTTTGGGGACGGCGAGGACACGCGGCGACCGGAGAGAGGCTACCTGACCCGCGCAGCCCGGACTCCCTTCCCTTCCCTTCCAGGGACGCCTGGTTTCCCCCCAAGCGAACCGGGATGGGAAGTGACTTCAATGAGATTGAACTTCAGCTGGATTGAAAGAGAGGCTAGAAGTTCCGCTTGCCAGCAGCCTCCTTAGTAGAGCGGAATGAGTAATACCCACACGGTGCTTGTCTCACTTCCCCATCCGCACCCGGCCCTCACCTGCTGTCACCTCGGCCTCCCACACCCGGTCCGCGCTCCCCGCCCTCTTCCTCGCGTAGAACCGTGGGATCCTAGGTGGCAGGACTCAGAGCTAAGGTATCCACAGGCCATGAATTCCTTCCTAAATGAGCGGTCATCGCCGTGCAGGACCTTAAGGCAAGAAGCATCGGCTGACAGATGTGATCTCTGAACCTGAT

[1] Ingolia NT, Ghaemmaghami S, Newman JR, Weissman JS. Genome-wide analysis in vivo of translation with nucleotide resolution using ribosome profiling. Science 2009;324:218-23.

[2] Schmidt EK, Clavarino G, Ceppi M, Pierre P. SUnSET, a nonradioactive method to monitor protein synthesis. Nature methods 2009;6:275-7.
